# Water availability positions auxin response maxima to determine plant regeneration fates

**DOI:** 10.1038/s41477-025-02029-2

**Published:** 2025-07-04

**Authors:** Abdul Kareem, Anna K. van Wüllen, Ai Zhang, Gabriel Walckiers, Ellen Fasth, Charles W. Melnyk

**Affiliations:** 1 Department of Plant Biology, Linnean Center for Plant Biology, Swedish University of Agricultural Sciences, Uppsala, Sweden; 2 College of Life Sciences, Northwest A&F University, Yangling, China; 3 Present Address: Louvain Institute of Biomolecular Science and Technology, UCLouvain, Louvain-la-Neuve, Belgium

**Keywords:** Plant regeneration, Cell fate

## Abstract

Wounding and hormones serve as diverse triggers for regeneration in animals and plants. Despite important advances in understanding various types of regeneration, the mechanism by which plants determine regeneration outcomes remains largely unknown. Here we demonstrate in *Arabidopsis* that a trade-off between two regeneration fates, wound-induced callus and root regeneration, was driven by distinct molecular pathways related to cambium and root development, respectively. We discovered that local water availability near the wound site determined the early stages of regeneration fates in *Arabidopsis* and tomato, with high water triggering root fate and low water initiating callus fate. Distinct spatial distributions of auxin response maxima around the wound, shaped by water availability, were critical for determining root or callus fates. We found that, by perturbing auxin response or auxin transport dynamics, we could change regeneration outcomes. Moreover, high water availability enhanced ethylene and jasmonic acid responses, whereas treatments with these hormones could modify auxin transport dynamics or the location of auxin response maxima, thus influencing regeneration fates. We propose that, through stress hormones, water availability modifies the auxin response distribution to control regeneration outcomes, thus allowing environmental control of regeneration and providing a means to improve in vitro regeneration by changing the water potential.

## Abstract (web summary)

This study reveals that water availability at the wound determines plant regeneration outcomes. High water availability induces root regeneration whereas low water availability triggers callus formation via stress hormones that shape auxin response maxima to activate distinct regeneration pathways.

## Main

Plants adapt to fluctuating environmental conditions, a remarkable trait largely controlled by their developmental plasticity. Such plasticity relies on cell fate determination by internal hormonal signals, transcription factors and receptors as well as the external environmental factors such as water availability, nutrient supply and temperature. For instance, roots use hydropatterning to form lateral roots in response to local water availability^1–3^. Similarly, a population of pericycle cells within roots display high plasticity to give rise to lateral roots, cambium or periderm depending on root developmental age and hormonal cues^4–6^. Such pericycle-derived cells express transcription factors such as *LATERAL ORGAN BOUNDARIES DOMAIN16* (*LBD16*) when obtaining lateral root fates^5,7^, whereas they express *LBD11* and *WUSCHEL‐RELATED HOMEOBOX4* (*WOX4*) when obtaining cambium fates^4,5,8,9^.

This developmental plasticity also underpins the regenerative capacity of plants, crucial for wound healing, biotechnology and plant propagation. Culturing plant tissues on hormone-rich medium induces pericycle cells to form pluripotent cell masses called callus that, by changing the ratio of auxin and cytokinin, can be subsequently regenerated into tissues such as shoots or roots^10–12^. Such hormone-induced callus (HIC) expresses root stem cell regulators *PLETHORA1* (*PLT1*) and *PLT2* and occurs via molecular pathways resembling root meristem development^13,14^. However, when plant tissues are wounded, they also trigger a regeneration process, for example, wound-induced callus (WIC) formation, de novo root regeneration (DNRR), root tip healing or vascular reconnection^15–20^. Such regeneration pathways allow plants to recover from injury without external hormonal requirement. With WIC, activation of transcription factors such as *WOUND INDUCED DEDIFFERENTIATION1* (*WIND1*) and *WOX13*, along with the peptide REGENERATION FACTOR1 (REF1) and hormone cytokinin, initiates callus formation, seals the wound and later differentiates to missing tissues^17,21–24^. During DNRR, auxin activates *WOX11* and *LBD16* transcription factors to facilitate adventitious root formation from wounded tissues^16,25,26^. Although the molecular players involved in different regeneration processes are well known, how plants determine whether to trigger a specific regeneration fate remains unknown. Moreover, unlike DNRR and HIC, the molecular fate of WIC is not well understood.

Here, by investigating diverse regeneration fates initiated from the same tissue, we demonstrated a regulatory interplay between different forms of regeneration driven by distinct cambium- or root-related molecular pathways. We discovered that water availability is a factor deciding whether plant tissues undergo root-mediated or callus-mediated regeneration fates in both *Arabidopsis* and tomato. Furthermore, we revealed that the spatial distribution of auxin response maxima is relevant for such fate changes and that water availability regulates such auxin maxima through the hormones ethylene and jasmonic acid.

## Results

### Different regeneration fates use distinct molecular pathways

**Fig. 1:**
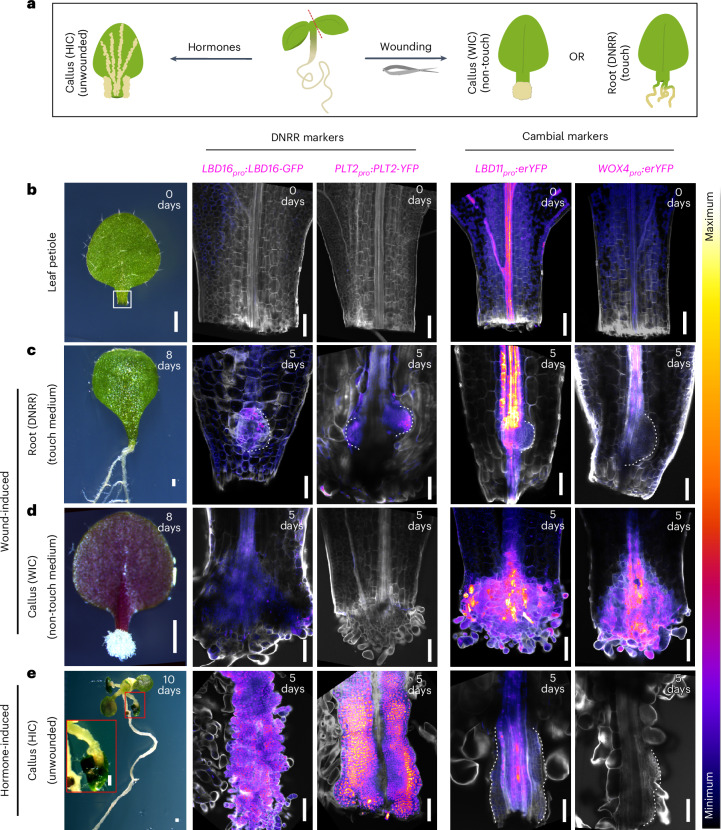
Different regeneration fates use distinct molecular pathways. **a**, A schematic showing diverse plant regeneration modes: HIC formation, WIC formation and wound-induced DNRR. **b**–**e**, Expression patterns of fluorescence reporters (magenta) for *LBD16*, *PLT2* (DNRR markers), *LBD11* and *WOX4* (cambial markers) in the *Arabidopsis* leaf petiole on day 0 (**b**) or during DNRR (**c**), WIC (**d**) or HIC from unwounded tissues (**e**) on day 5. Dashed lines indicate the regenerating root primordium in **c** and callus in **e**. Calcofluor-white (grey) was used to stain cell walls. The corresponding representative bright-field images of the detached leaf (**b**), DNRR (**c**), WIC (**d**) and HIC (**e**) at 0, 8 and 10 days are shown on the left. A magnified view of the callus from unwounded petiole, outlined by a red box, is shown as an inset in the bright-field image of **e**. Scale bars, 500 µm (bright-field images) and 100 µm (confocal images). Confocal experiments were independently performed three times with similar results.

**Extended Data Fig. 1:**
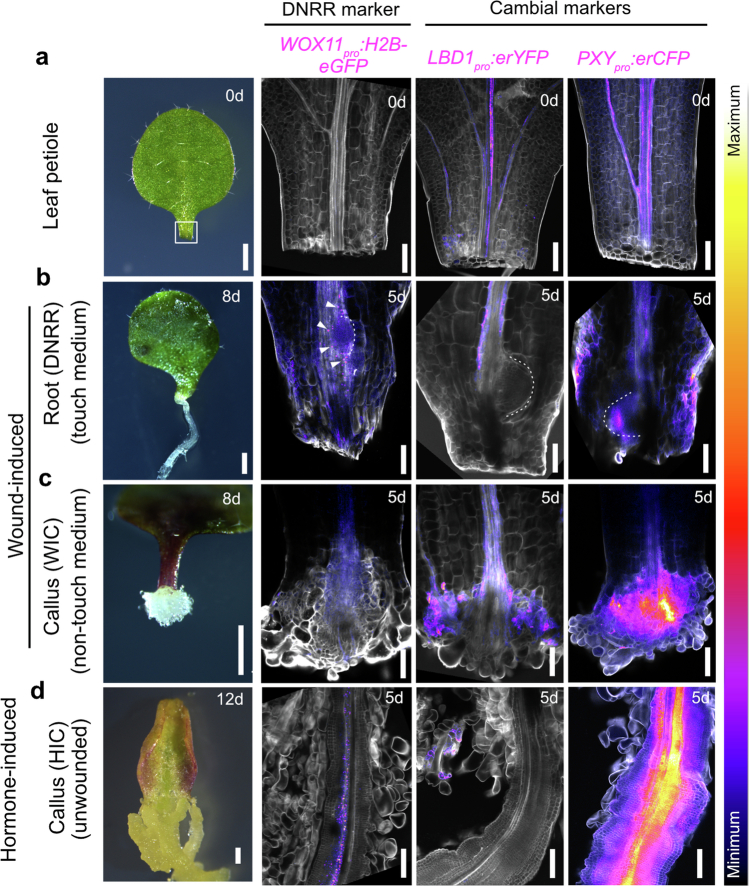
Wound-induced callus activates cambium-related markers but not root regeneration-related markers. **a**–**d**, Expression patterns of fluorescence reporters (magenta) for *WOX11* (*de novo* root regeneration (DNRR) marker)*, LBD1* and *PXY* (cambial markers) in the *Arabidopsis* leaf petiole on day 0 (**a**) or during DNRR (**b**), wound-induced callus (WIC) (**c**), or hormone-induced callus (HIC) from unwounded tissues (**d**) on day 5. Dashed lines indicate the regenerating root primordium in (**b**). Calcofluor white (grey) was used to stain cell walls. The corresponding representative bright-field images of the detached leaf (**a**), DNRR (**b**), WIC (**c**) and HIC (**d**) at 0, 8 or 12 days are shown in the left panel. Scale bar: 500 µm for the bright-field images, 100 µm for the confocal images. Confocal experiments were independently performed 3 times with similar results.

**Extended Data Fig. 2:**
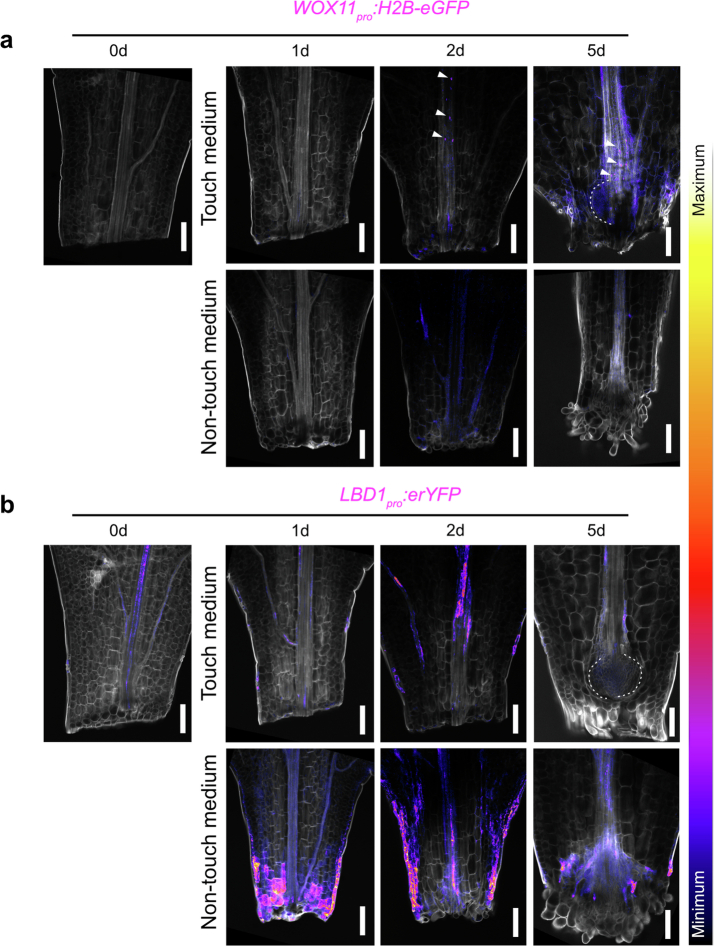
Spatio-temporal expression pattern of DNRR and WIC marker genes. **a**, **b**, Expression patterns of fluorescence reporters (magenta) for *WOX11* (*de novo* root regeneration (DNRR) marker) (**a**) and *LBD1* (cambial marker) (**b**) in the *Arabidopsis* leaf petiole. Images were taken on day 0 and at time points of 1, 2, and 5 days during DNRR (touch medium) or wound-induced callus (WIC) (non-touch medium) formation. Arrowheads mark the *WOX11* signal in (**a**). Dashed lines indicate the regenerating root primordium. Calcofluor white (grey) was used to stain cell walls. Scale bar:100 µm. Confocal experiments were independently performed 3 times with similar results.

The same plant tissue can exhibit different types of regeneration, yet previous studies have used different tissues undergoing WIC formation, wound-induced DNRR or HIC formation^14,16,17,23,27–29^. To more accurately compare these processes, we used a single tissue, the *Arabidopsis* leaf petiole, and sought to determine whether different regeneration fates had common or distinct molecular pathways. DNRR was initiated at the wound site of the petiole touching hormone-free culture medium, while WIC was induced at the wound site when not in contact with the medium (Fig. 1a–d), consistent with previous findings^15^. HIC, meanwhile, was triggered by culturing unwounded tissues in an auxin-rich medium (Fig. 1e). We focused on key genes implicated in DNRR and observed activation of *LBD16*, *PLT2* and *WOX11* during both DNRR and HIC, while their expression was low in WIC (Fig. 1b–e and Extended Data Figs. 1a–d and 2a). We monitored the expression patterns of genes associated with the vascular cambium and found *LBD1*, *LBD11*, *PHLOEM INTERCALATED WITH XYLEM* (*PXY*) and *WOX4* activated in the proliferating cells of WIC. Their expression levels were generally lower during both DNRR and HIC except for *PXY*, which was also induced in HIC (Fig. 1c–e and Extended Data Figs. 1b–d and 2b). The similar expression profiles of DNRR and unwounded HIC suggest that they probably shared similar molecular pathways, consistent with the finding that HIC from wounded tissues follows a root meristem pathway^14^, while WIC probably used a pathway linked to the vascular cambium.

### WIC inhibits root regeneration

**Fig. 2:**
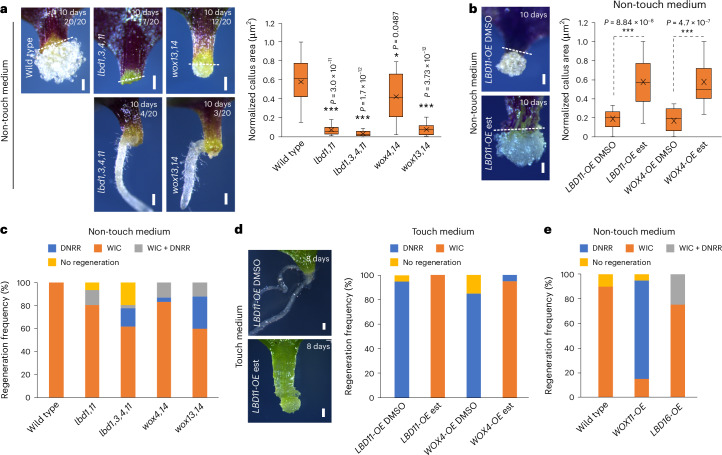
WIC inhibits root regeneration. **a**, Top: WIC formation in leaf petiole explants of *Arabidopsis* wild-type and cambial mutants, with the wound site not touching the medium. Bottom: DNRR in the mutants under the same conditions. Numbers on the image indicate how many plants out of 20 showed the phenotype. Normalized WIC area quantifications of wild-type and mutant explants are shown to the right (*n* = 21, 21, 21, 21 and 24 (left to right) independent explants; *P < 0.05, ***P < 0.001; two-tailed Student’s *t*-test; box plot shows median (central line), mean (cross), first and third quartiles (top and bottom edges), and minimum and maximum values excluding outliers (whiskers)) **b**, WIC formation images or quantifications in the cotyledon explants of *LBD11-overexpression* (OE) or *WOX4-OE* with the wound site not touching the medium. Mock (DMSO) and 5 µM oestradiol (est) treatments are shown (*n* = 19, 20, 17 and 15 (left to right) independent explants; ***P < 0.001; two-tailed Student’s *t*-test; box plot details as in **a**). **c**, The frequency of regeneration of WIC and DNRR in the leaf petioles of various mutants with the wound site not in contact with the medium (*n* = 41, 36, 44, 41 and 45 (left to right) independent explants, mean). **d**, WIC formation images or quantifications in the leaf explants of *LBD11-OE* or *WOX4-OE* when the wound site is in contact with the medium. Mock (DMSO) and 5 µM oestradiol (est) treatments are shown (*n* = 20 independent explants per genotype; mean). **e**, Regeneration frequencies of DNRR and WIC in the leaf petiole of *LBD16-OE* (constitutive) and *WOX11-OE* (constitutive) with the wound site not touching the medium (*n* = 20 independent explants per genotype; mean). Dashed lines indicate the wound site in **a** and **b**. Scale bar, 200 µm.

**Extended Data Fig. 3:**
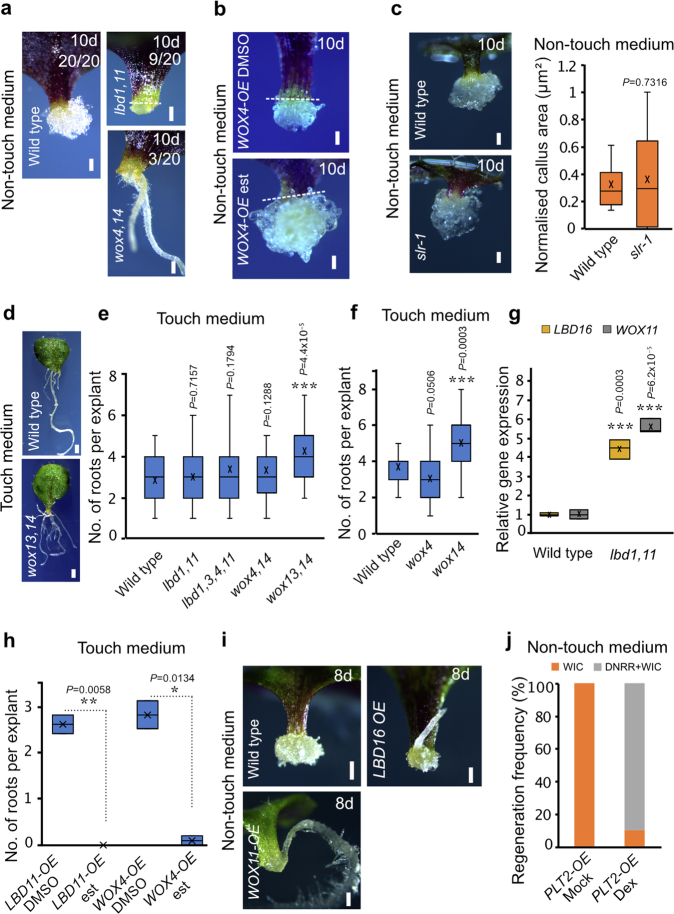
A trade-off between wound-induced callus and root regeneration determines regeneration fates. **a**, Formation of wound-induced callus (WIC) or *de novo* root regeneration (DNRR) in leaf explants of wild type and cambial mutants with wound sites not in contact with the medium. **b**, WIC formation in the cotyledon explants of *WOX4-OE* with the wound site not touching the medium. **c**, Images and quantifications of normalized WIC areas of wild type and *slr-1* cotyledon petioles with the wound site not touching the medium (n = 18 (wild type), 16 (*slr-1*) independent explants; two-tailed Student’s t-test; box plot shows median (central line), mean (cross), first and third quartiles (top and bottom edges), minimum and maximum values excluding outliers (whiskers)). **d**, Representative images showing DNRR in the *wox13,14* mutant with the wound site touching the medium. **e**, **f**, Frequency of DNRR in various mutants compared to wild type with the wound site touching the medium. (n = 29, 31, 28, 28, 29 (left to right) independent explants in (**e**) and 35, 24, 27 (left to right) independent explants in (**f**); ***P ≤ 0.001; two-tailed Student’s t-test; box plot details as in (**c**)). **g**, *LBD16* and *WOX11* transcript levels in *lbd1,11* mutant petioles 4 days post-wounding with the wound site not touching the medium, measured by RT-qPCR. Expression normalized to ACTIN2. (n = 3 independent biological replicates with 3 technical replicates each; ***P < 0.001; two-tailed Student’s t-test; box plot details as in (**c**)). **h**, Frequency of DNRR in *WOX4-OE* and *LBD11-OE* with the wound site touching the medium. (n = 20 independent explants per genotype; *P < 0.05; **P < 0.01; two-tailed Student’s t-test; box plot details as in (**c**)). **i**, DNRR formation in *LBD16-OE* (constitutive) and *WOX11-OE* (constitutive) with the wound site not touching the medium. **j**, DNRR and WIC formation frequencies in the petiole of *PLT2-OE* with the wound site not touching the medium. Mock (ethanol) and 5 µM dexamethasone (DEX) treatments are shown (n = 10 independent explants per genotype; mean). Scale bar: 500 µm in (**d**) and 200 µm in the rest.

To further investigate the molecular basis for WIC, we tested cambial mutants, including *lbd1,11*; *lbd1,3,4,11*; *wox4,14*; and *wox13,14*, and found them to be defective in WIC formation (Fig. 2a and Extended Data Fig. 3a), suggesting an essential role of cambial genes in this process. Inducible overexpression of *LBD11* or *WOX4* also boosted WIC formation (Fig. 2b and Extended Data Fig. 3b). By contrast, the pericycle cell division-defective mutant *solitary root1* (*slr-1*) did not inhibit WIC formation (Extended Data Fig. 3c). Despite deficient WIC formation, cambial mutants displayed an enhanced ability to form DNRR, even in conditions where wild-type tissues failed to form roots (Fig. 2a,c and Extended Data Fig. 3a,d–f). We observed increased DNRR-related gene transcription in the WIC deficient *lbd1,11* mutant (Extended Data Fig. 3g), implying suppression of WIC activated DNRR-related pathways. In addition, overexpression of WIC-promoting genes *LBD11* and *WOX4* inhibited the DNRR pathway, while overexpression of DNRR-promoting genes *LBD16*, *PLT2* and *WOX11* suppressed the WIC pathway (Fig. 2d,e and Extended Data Fig. 3h–j). Our data indicate that activation of cambial cells was crucial for WIC formation and that WIC fate inhibited DNRR fate, suggesting a trade-off between regeneration fates.

### Water availability determines regeneration fates

**Fig. 3:**
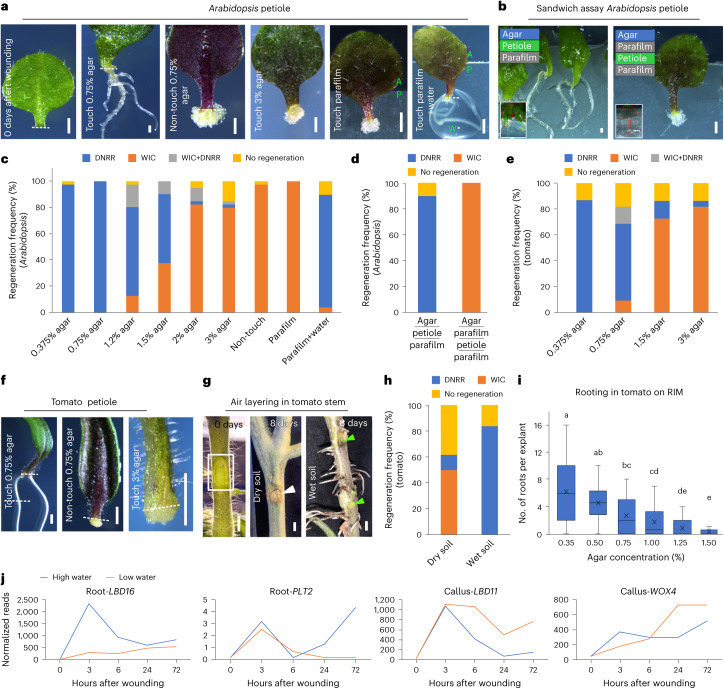
The trade-off between regeneration of callus and root is determined by the availability of water. **a**, Root or callus regeneration in *Arabidopsis* leaf petioles with wound sites either touching or non-touching on media with different agar concentrations, or on parafilm with or without water (10 days after wounding). The dashed line marks the wound site. A, agar; P, parafilm; W, water. **b**, Root or callus regeneration in *Arabidopsis* leaf petioles placed on parafilm–agar sandwich set-ups: parafilm–petiole–agar or parafilm–petiole–parafilm–agar (10 days after wounding). Insets show the leaf (dashed red line) in the set-up. **c**, The regeneration frequency in *Arabidopsis* leaf petioles under varying water availability (*n* = 39, 40, 40, 40, 39, 39, 40, 40 and 28 (left to right) independent explants; mean). **d**, The regeneration frequency in *Arabidopsis* leaf petioles under sandwich set-ups (*n* = 20 independent explants per condition; mean). Underlining indicates the order of layers. **e**, The regeneration frequency in tomato cotyledon petioles under different agar conditions (*n* = 22 independent explants per condition; mean). **f**, Regeneration in tomato cotyledon petioles on 0.75% or 3% agar, 10 days after wounding. The dashed line marks the wound site. **g**,**h**, Images (**g**) and regeneration frequency (**h**) in tomato stems during air layering with wet or dry soil (*n* = 18 independent explants per condition; mean). Wound sites are marked by a rectangle (0 days) and green arrowheads (8 days wet soil) and calli are marked by a white arrowhead. **i**, The average number of regenerated roots per tomato cotyledon explant on auxin-containing (0.5 mg l^−1^ indole-3-acetic acid, IAA) RIM with varying agar concentrations (*n* = 19, 18, 19, 18, 19 and 17 (left to right) independent explants; letters indicate significant differences (*P* ≤ 0.05), Kruskal–Wallis with pairwise Wilcoxon; box plot shows median (central line), mean (cross), first and third quartiles (top and bottom edges), and minimum and maximum values excluding outliers (whiskers)). **j**, The transcriptional dynamics of genes associated with callus and root regeneration in *Arabidopsis*. Scale bars, 500 µm (**a** and **b**), 1 mm (**f**) and 5 mm (**g**).

**Extended Data Fig. 4:**
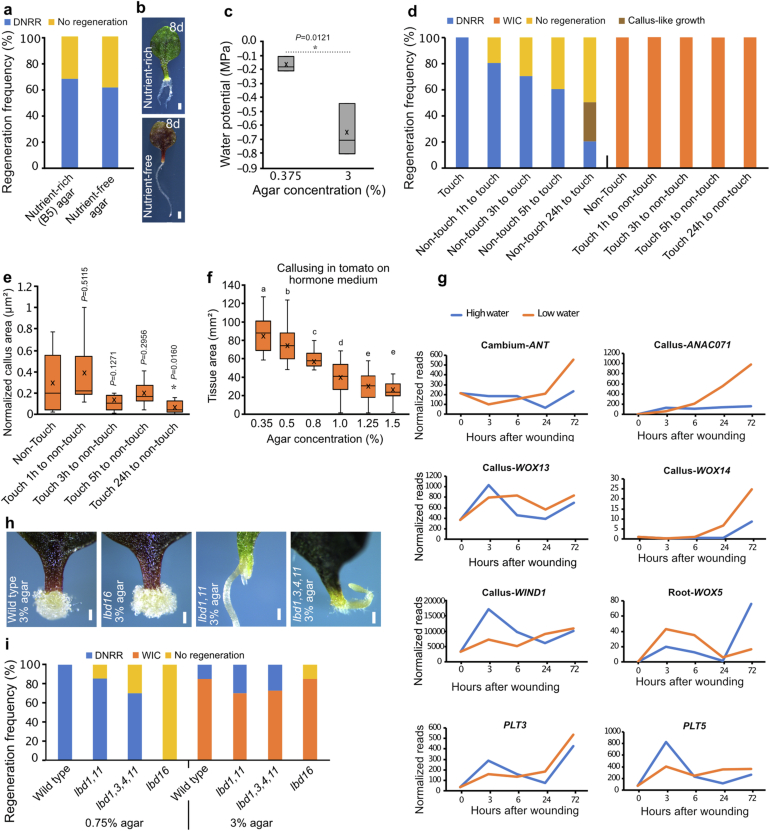
Water availability determines regeneration fates. **a**, Regeneration frequencies in *Arabidopsis* leaf petioles with the wound site touching either nutrient rich or nutrient-free agar medium 8 days post wounding. (n = 19 (nutrient-rich),18 (nutrient-free) independent explants; mean). **b**, Root regeneration in petioles exposed to either nutrient-rich or nutrient-free agar media. **c**, Correlation between water potential (MPa) and agar concentration of B5 medium. (n = 3 independent biological replicates with 3 technical replicates each; *P < 0.05; two-tailed Student’s t-test; box plot shows median (central line), mean (cross), first and third quartiles (top and bottom edges), minimum and maximum values excluding outliers (whiskers)). **d**, Regeneration frequencies assessed 10 days after wounding by altering tissue orientation (touch to non-touch or vice versa) for different durations in *Arabidopsis* petioles. (n = 10 independent explants per treatment; mean). **e**, Normalized wound-induced callus area in petioles after altering tissue orientation from touch to non-touch for different durations (n = 10 independent explants per treatment; *P < 0.05; two-tailed Student’s t-test; box plot details as in (**c**)). **f**, Hormone-induced callus area in tomato cotyledon explant cultured on callus inducing medium (CIM) under varying agar concentrations. (n = 23, 24, 23, 24, 26, 23 (left to right) independent explants; letters indicate significant differences (*P* ≤ 0.05); Kruskal–Wallis with pairwise Wilcoxon; box plot details as in (**c**)). **g**, Transcriptional dynamics of genes associated with cambium, callus and root at high (0.75% agar) and low (2% agar) water conditions. **h**, Wound-induced callus (WIC) or *de novo* root regeneration (DNRR) in cambial and DNRR mutants under low water (3% agar) conditions. **i**, Regeneration frequencies in cambial and DNRR mutants under high (0.75% agar) and low (3% agar) water conditions. (n = 20, 21, 10, 20, 20, 20, 11, 20 (left to right) independent explants; mean). Scale bar: 500 µm in (b), 200 µm in (**h**).

**Extended Data Fig. 5:**
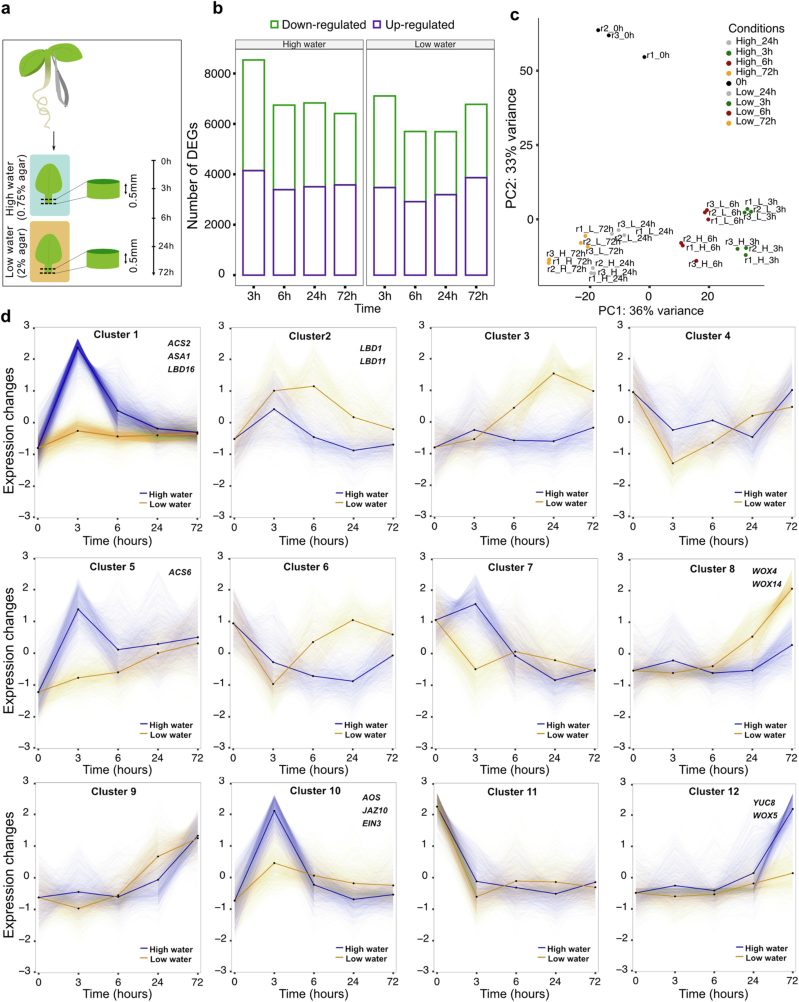
Transcriptional dynamics of regeneration fate changes. **a**, Schematic depicting 0.5 mm tissue harvesting and time points for *Arabidopsis* petiole wound sites under high and low water conditions for RNA-seq. **b**, The distribution of differentially expressed genes (DEGs) in response to high and low water conditions. **c**, Principal Component Analysis (PCA) of the gene expression data from the regeneration fate transcriptome. The two principal components (PC1 and PC2) explain 69% of the total variation in the regeneration transcriptome. Different colors indicate various time points after wounding (in hours). Data from three biological replicates (r1, r2, r3) per water condition (high (H) or low (L) water) per time point are shown. **d**, Clustering analysis of transcriptional dynamics under high and low water availability conditions (12 clusters in total). Lines represent the average expression levels of DEGs within each cluster. Representative genes for selected clusters are shown in the top right corner.

What determined cell fate was unknown, but we observed that, when the wound site was in contact with B5 agar medium, DNRR was triggered (Fig. 3a,c), whereas contact with parafilm or air favoured WIC formation (Fig. 3a,c) Similarly, DNRR was initiated when the petiole was sandwiched between agar and parafilm, whereas WIC formed when the petiole was sandwiched between two layers of parafilm, suggesting that physical contact or force alone did not determine regeneration fates (Fig. 3b,d). Nutrient availability also had no substantial impact (Extended Data Fig. 4a,b). We then tested the role of water by culturing leaf tissues with the wound site in contact with B5 medium with various agar concentrations (0.375% to 3%) that represented different water potentials (Extended Data Fig. 4c). This variation corresponded to the available water at the medium surface, as previously described^3^. We found that higher water availability (lower agar concentrations) favoured DNRR, whereas lower water availability (higher agar concentrations) promoted WIC (Fig. 3a,c). Applying water to the wound site touching parafilm induced DNRR, whereas parafilm alone led to WIC formation (Fig. 3a,c). This fate decision was made shortly after wounding because changes in water availability during this period impacted regeneration outcomes (Extended Data Fig. 4d,e). Similar to *Arabidopsis*, tomato tissues also responded to water availability by regenerating roots or calli (Fig. 3e,f). Air layering is commonly used to propagate plants, so we used this technique on tomato stems and found that damp soil promoted DNRR whereas dry soil promoted WIC (Fig. 3g,h). This supports the observation that water availability, rather than physical contact with the soil, was the primary factor determining regeneration outcomes. In addition, we found that water availability had a substantial impact on root regeneration and callus formation rates in tomato cotyledon explants cultured on hormone-containing root-inducing medium (RIM) and callus-inducing medium (CIM), respectively (Fig. 3i and Extended Data Fig. 4f). To further understand the genes involved in fate decision, we conducted RNA sequencing (RNA-seq) on wounded *Arabidopsis* petioles at various timepoints with high water (0.75% agar) or low water (2% agar) concentrations (Extended Data Fig. 5a–d). DNRR-promoting genes (*LBD16*, *PLT2* and *WOX5*) were predominantly induced under high-water conditions, while WIC-promoting genes (*LBD11*, *WOX4* and *WOX14*) were primarily upregulated under low-water conditions, although there was some variation at different timepoints (Fig. 3j and Extended Data Fig. 4g). The corresponding mutants displayed defects in either WIC or DNRR, consistent with a role for promoting either DNRR or WIC during varying water availability (Extended Data Fig. 4h,i). Overall, our findings suggest that water availability at the wound site was crucial for deciding whether tissues undergo root or callus regeneration in *Arabidopsis* and tomato.

### Water availability regulates stress hormones in regeneration

**Fig. 4:**
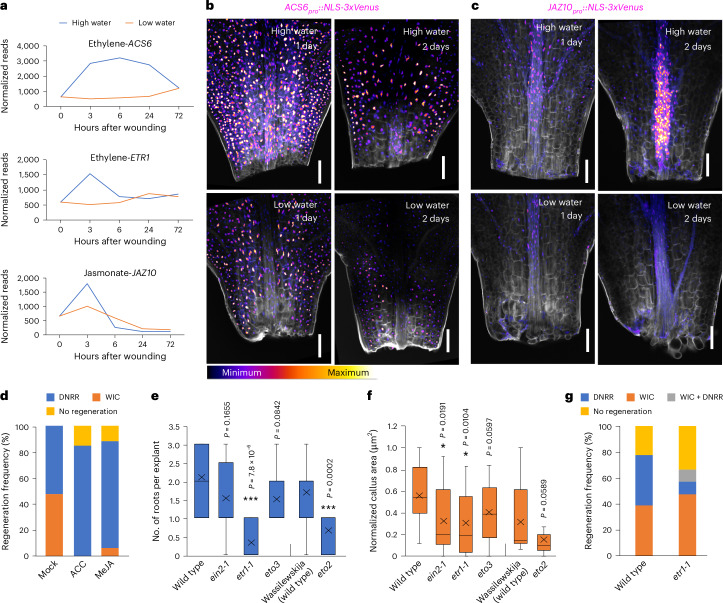
Water availability regulates stress hormones. **a**, Transcriptional dynamics of genes associated with ethylene and jasmonic acid synthesis and signalling. **b**,**c**, Ethylene biosynthesis reporter *ACS6-VENUS* (**b**) and jasmonic acid-response reporter *JAZ10-VENUS* (**c**) expression under conditions of high (0.75% agar) and low (2% agar) water availability. **d**, Regeneration frequencies after treatment with ethylene precursor (4 µM ACC) or jasmonic acid (5 µM MeJA) under intermediate-water conditions (1.5% agar) (*n* = 19, 20 and 17 (left to right) independent explants; mean). **e**, The frequency of root regeneration in ethylene mutants compared with wild type under high-water conditions (*n* = 10, 13, 12, 12, 16 and 12 (left to right) independent explants; ***P < 0.001; two-tailed Student’s *t*-test; box plot shows median (central line), mean (cross), first and third quartiles (top and bottom edges), and minimum and maximum values excluding outliers (whiskers)). **f**, The normalized WIC area of wild type and ethylene mutants under low-water conditions (*n* = 18, 19, 19, 25, 20 and 21 (left to right) independent explants; *P < 0.05; two-tailed Student’s *t*-test; box plot details as in **e**). **g**, The regeneration frequency in the *etr1-1* mutant under intermediate-water conditions (*n* = 18 (wild type), 21 (*etr1-1*) independent explants, mean). Scale bar, 100 µm. The look-up table in **b** displays the signal intensity range used for **b** and **c**. Confocal experiments (**b** and **c**) were independently performed three times with similar results.

**Extended Data Fig. 6:**
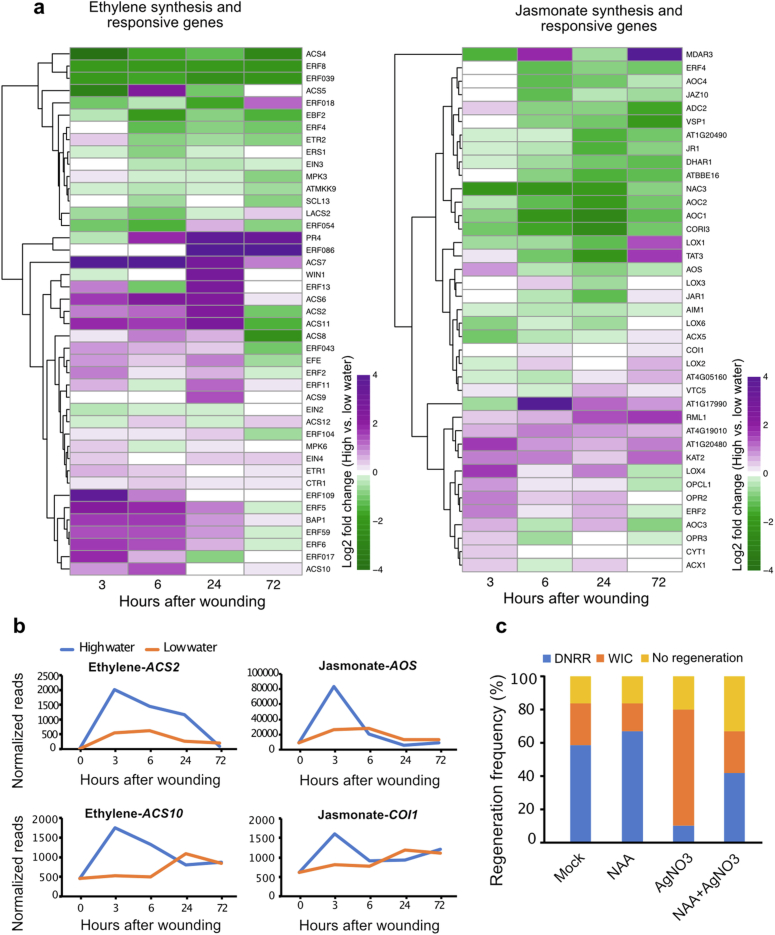
Water availability influences expression of ethylene and jasmonic acid biosynthesis and response genes. **a**, Heatmaps showing fold changes of expression of genes involved in ethylene, and jasmonic acid synthesis and response under high (0.75% agar) vs. low (2% agar) water conditions in wounded *Arabidopsis* petioles. **b**, Transcriptional dynamics of genes associated with ethylene and jasmonic acid synthesis and signalling. **c**, Regeneration frequencies after treatments with 0.2 µM NAA, 10 µM AgNO3 or NAA+AgNO3 at intermediate water conditions. (n = 12, 18, 10, 12 (left to right) independent explants; mean). DNRR= *de novo* root regeneration, WIC=wound-induced callus.

Stress hormones are important for wound response^28,30^ and water availability response^31^, so we analysed our RNA-seq dataset for hormone-responsive genes. We observed activation of genes responsive to ethylene and jasmonic acid in our RNA-seq datasets within 3 h of wounding under high-water conditions (Fig. 4a and Extended Data Fig. 6a,b). Reporter gene analyses also showed upregulation of ethylene biosynthesis (*ACS6-VENUS*) and jasmonic acid response (*JAZ10-VENUS*) markers in high-water conditions (Fig. 4b,c). Consistent with a role for these hormones, short treatments of ethylene precursor (4 µM 1-aminocyclopropane-1-carboxylate, ACC) and jasmonic acid (5 µM methyl-jasmonate, MeJA) enhanced the DNRR-to-WIC ratio (Fig. 4d). Similarly, treatments with the ethylene inhibitor silver nitrate suppressed DNRR fate and promoted WIC fate, although the resulting callus area was smaller. This effect was partially rescued by auxin (1-naphthaleneacetic acid, NAA) treatment (Extended Data Fig. 6c). We next analysed loss-of-function mutants and found that the ethylene signalling mutant *ethylene resistant1-1* (*etr1-1*)^32^ exhibited defects in both DNRR and WIC (Fig. 4e,f). However, it displayed a greater impairment in the DNRR fate, leading to a higher WIC-to-DNRR ratio at intermediate water availability (Fig. 4g). Notably, the ethylene overproducing mutant *eto2*^33^ also affected DNRR, with minimal impact on WIC (Fig. 4e,f). Although our transcriptome and treatment assays indicated that increases in ethylene and jasmonic acid promoted DNRR, a basal level of ethylene signalling appeared relevant for both DNRR and WIC (Fig. 4e,f).

### Water availability shapes spatial auxin response

**Fig. 5:**
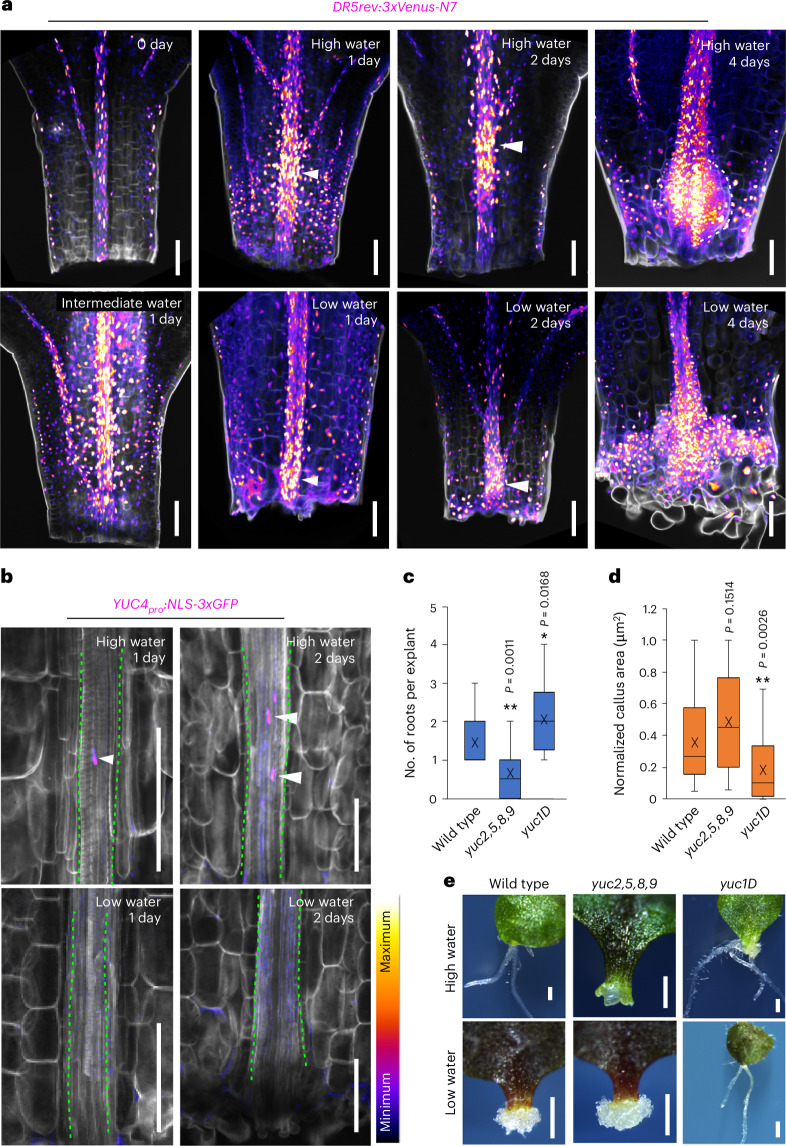
Water availability shapes spatial auxin response during regeneration. **a**, *DR5-VENUS* auxin-responsive reporter under conditions of high (0.75% agar), intermediate (1.5% agar) and low (2% agar) water availability, with arrowheads indicating auxin maxima in the *Arabidopsis* leaf petiole. **b**, The expression of *YUC4–GFP* under conditions of high (0.75% agar) and low (2% agar) water availability. Dashed green lines mark the leaf vasculature, and arrowheads highlight *YUC4–GFP* signal. **c**, The average amount of root regeneration per petiole explant in wild type, *yuc2,5,8,9* and *yuc1D* under high-water conditions (*n* = 20 independent explants per genotype; *P < 0.05, **P < 0.01, two-tailed Student’s *t*-test; the box plot shows median (central line), mean (cross), first and third quartiles (top and bottom edges), and minimum and maximum values excluding outliers (whiskers)). **d**, The normalized WIC area in *yuc1D* and *yuc2,5,8,9* (*n* = 19, 20 and 15 (left to right) independent explants; **P < 0.01, two-tailed Student’s *t*-test; box plot details as in **c**). **e**, Representative images showing WIC and DNRR in *yuc1D* and *yuc2,5,8,9* mutants under conditions of high (0.75% agar) and low (2% agar) water availability. Scale bars, 100 µm (**a** and **b**) and 500 µm (**e**). The look-up table in **b** displays the signal intensity range used for **a** and **b**. Confocal experiments (**a** and **b**) were independently performed three times with similar results.

**Extended Data Fig. 7:**
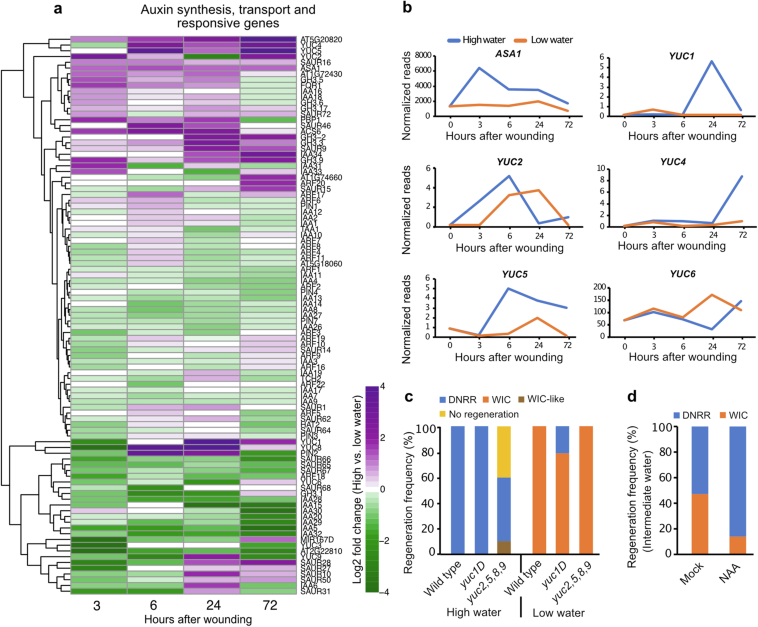
Water availability influences expression of auxin biosynthesis, transport and responsive genes during regeneration. **a**, Heatmap showing fold changes of expression of genes involved in auxin synthesis, transport and response under high (0.75% agar) vs. low (2% agar) water conditions in wounded *Arabidopsis* petioles. **b**, Transcriptional dynamics of auxin biosynthesis genes at high and low water conditions. **c**, Regeneration frequencies in *yuc1D* and *yuc2,5,8,9* mutants at high and low water conditions (n = 20, 20, 20, 19, 19, 20 (left to right) independent explants; mean). **d**, Regeneration frequencies after 0.2 µM NAA treatment under intermediate water conditions (1.5% agar). (n = 19 (mock), 14 (NAA) independent explants; mean). DNRR= *de novo* root regeneration, WIC=wound-induced callus.

We further analysed the hormone response, and our transcriptome data revealed differential expression of many auxin-responsive genes under varying water availability (Extended Data Fig. 7a). We therefore explored auxin response distribution using the auxin-responsive reporter DR5-VENUS and observed distinct spatial expression patterns in *Arabidopsis* tissues across water conditions. High water availability (0.75% agar) caused the auxin maxima to form distal to the wound at the root primordium initiation site, whereas low water availability (2% agar) caused it to form near the wound site (Fig. 5a). Intermediate water availability (1.5% agar), promoting both DNRR and WIC regeneration, resulted in a broader auxin distribution (Fig. 5a).

**Extended Data Fig. 8:**
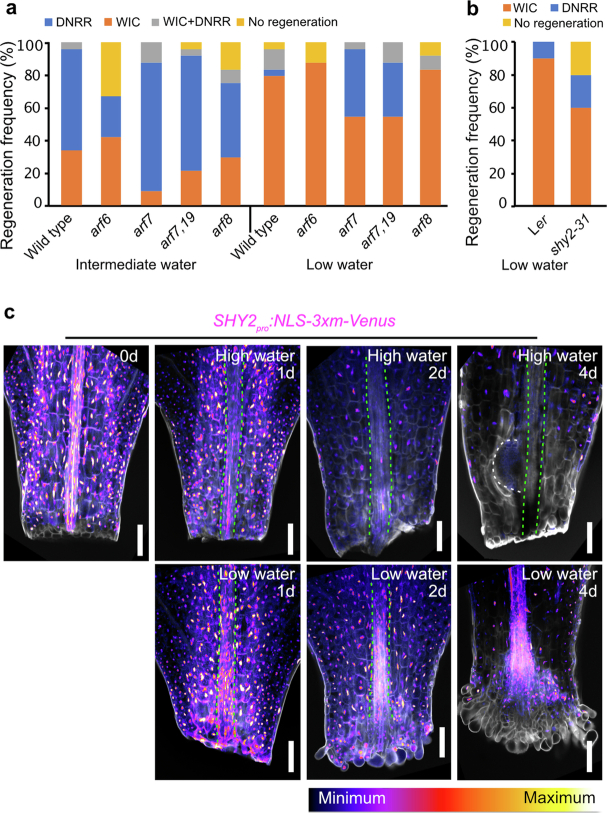
Auxin signalling is involved in distinct regeneration pathways. **a**, Regeneration frequencies in *arf6*, *arf7* and *arf8* mutants at intermediate (1.5% agar) and low (2% agar) water conditions (n = 24 independent explants per genotype; mean). **b**, Regeneration frequencies in the *shy2-31* mutant under low water conditions (3% agar) (n = 10 independent explants per genotype). **c**, Spatial expression of *SHY2-VENUS* under high (0.75% agar) and low (2% agar) water conditions. Green dotted lines mark vasculature, while the white dotted line marks the root primordium. DNRR= *de novo* root regeneration, WIC=wound-induced callus. Scale bar: 100 µm. Confocal experiments (**c**) were independently performed 3 times with similar results.

Auxin biosynthesis genes such as *ANTHRANILATE SYNTHASEα1* (*ASA1*), *YUCCA1* (*YUC1*), *YUC4* and *YUC5* were upregulated in high water, with *YUC4–GFP* activating specifically at the root primordium initiation site (Fig. 5b and Extended Data Fig. 7b). We tested a role for auxin biosynthesis and found the *yuc2,5,8,9* mutant to have impaired DNRR but normal WIC formation. Treatment with exogenous auxin or using the auxin-overproducing line *yuc1D* perturbed WIC but enhanced DNRR (Fig. 5c–e and Extended Data Fig. 7c,d). We investigated downstream auxin signalling and found that mutants *auxin response factor6* (*arf6*) and *arf8* displayed defects in DNRR, while *arf7* and *arf7,19* mutants showed an increased ratio of DNRR to WIC formation (Extended Data Fig. 8a). Similarly, the loss-of-function *SHORT HYPOCOTYL2* mutant, *shy2-31*, promoted DNRR under low water, indicating that high ARF7, ARF19 or SHY2 activity inhibits DNRR under low water (Extended Data Fig. 8b). A SHY2-VENUS reporter showed low signal in high water and high signal in low water (Extended Data Fig. 8c), consistent with its role as a negative regulator of DNRR in water conditions. Thus, auxin biosynthesis and signalling played an important role in fate decision, potentially operating through pathways involving DNRR-promoting factors and WIC-promoting factors.

### Stress hormones influence auxin response maxima

**Fig. 6:**
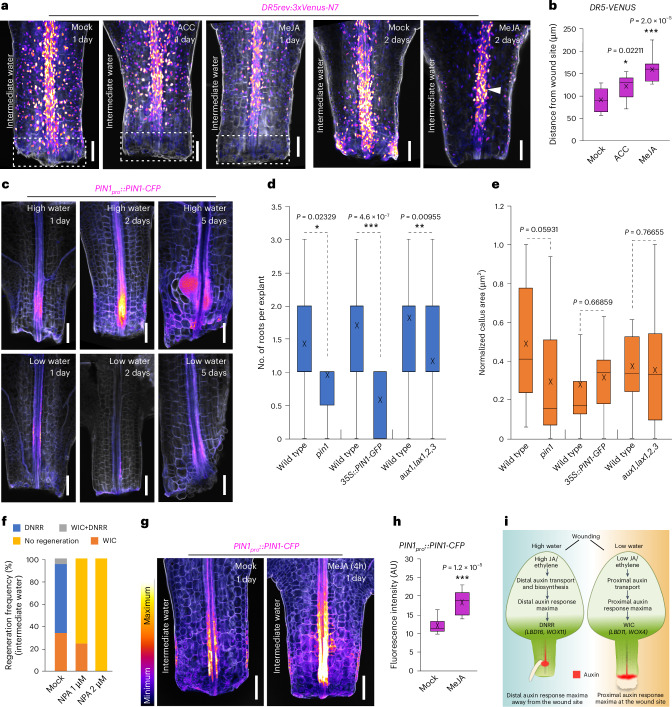
Stress hormones and auxin transport influence auxin response maxima. **a**, Changes in *DR5-VENUS* expression (white box) 1 day after treatment with 4 µM ACC (ethylene precursor) or 5 µM MeJA (jasmonic acid) under intermediate-water conditions (1.5% agar) in *Arabidopsis* petioles. After 2 days of 5 µM MeJA treatment, the *DR5-VENUS* signal becomes confined to the root primordia initiation site (arrowhead). **b**, Changes in *DR5-VENUS* expression domain measured as the distance from the wound site to the high *DR5-VENUS* signal, 1 day after 4 µM ACC or 5 µM MeJA treatment (*n* = 10, 10 and 11 (left to right) independent explants; *P < 0.05, ***P < 0.001, two-sided Student’s *t*-test; box plot shows median (central line), mean (cross), first and third quartiles (top and bottom edges), and minimum and maximum values excluding outliers (whiskers)). **c**, The expression pattern of *PIN1–CFP* under conditions of high (0.75% agar) and low (2% agar) water availability. Imaging was independently performed three times with similar results. **d**, The average amount of root regeneration per petiole explant in wild type, *pin1*, *35S::PIN1–GFP* and *aux1,lax1,2,3* under high-water conditions (*n* = 20, 20, 19, 21, 20 and 20 (left to right) independent explants; *P < 0.05, **P < 0.01, ***P < 0.001; two-tailed Student’s *t*-test; box plot details as in **b**). **e**, The normalized WIC area in wild type, *pin1, 35S::PIN1–GFP* and *aux1,lax1,2,3* under low-water conditions (*n* = 20, 19, 20, 19, 20 and 28 (left to right) independent explants; two-tailed Student’s *t*-test; box plot details as in **b**). **f**, Regeneration frequencies after NPA treatment (1 µM and 2 µM) under conditions of intermediate (1.5% agar) water availability (*n* = 20 independent explants per treatment). **g**, *PIN1–CFP* expression after mock or 5 µM MeJA treatment for 4 h under intermediate-water conditions (1.5% agar) and imaged 1 day after wounding. **h**, The average fluorescence intensity of *PIN1–CFP* after treatment with 5 µM MeJA or mock for 4 h under intermediate-water conditions (1.5% agar) (*n* = 10 independent explants per treatment; ***P < 0.001; two-tailed Student’s *t*-test; box plot details as in **b**). **i**, The working model showing that water availability determines regeneration fates in detached leaf tissues. High water triggers DNRR, while low water promotes WIC formation by shifting auxin response maxima through ethylene and jasmonic acid signalling. Scale bar, 100 µm. The look-up table in **g** displays the signal intensity range used for **a**, **c** and **g**.

**Extended Data Fig. 9:**
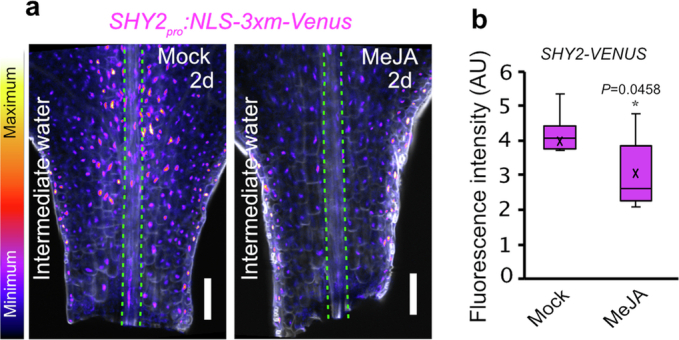
Jasmonic acid affects *SHY2* expression. **a**, *SHY2-VENUS* expression after mock or 5 µM MeJA treatment for 43 h at intermediate water conditions (1.5% agar). Green dotted lines mark vasculature. **b**, Average fluorescence intensity of *SHY2-VENUS* post-treatment with 5 µM MeJA or mock for 43 h at intermediate water conditions (1.5% agar). (n = 10 (mock), 9 (MeJA) independent explants; *P < 0.05; two-tailed Student’s t-test; box plot shows median (central line), mean (cross), first and third quartiles (top and bottom edges), minimum and maximum values excluding outliers (whiskers)). Scale bar: 100 µm.

**Extended Data Fig. 10:**
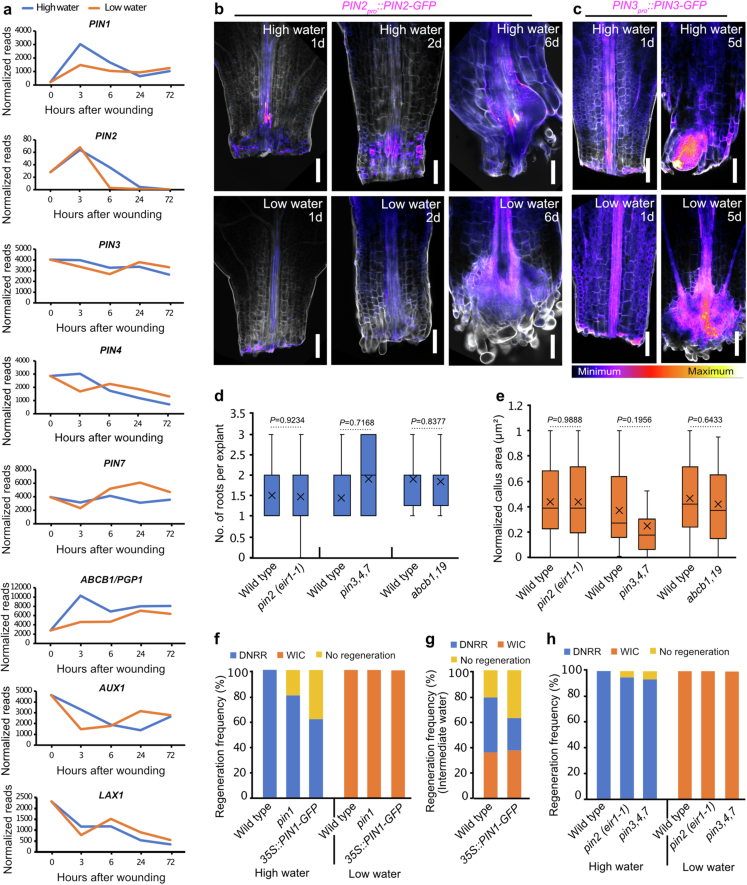
Water availability influences auxin transport during regeneration. **a**, Transcriptional dynamics of auxin transport genes at high and low water conditions. **b**, **c**, Expression pattern of *PIN2-GFP* (**b**) and *PIN3-GFP* (**c**) under high (0.75% agar) and low (2% agar) water conditions in the leaf petiole. **d**, Average number of root regeneration per petiole explant in wild type, *pin2, pin3,4,7* and *abcb1,19* under high water conditions (n = 10, 15, 20, 20, 20 20 (left to right) independent explants; two-tailed Student’s t-test; box plot shows median (central line), mean (cross), first and third quartiles (top and bottom edges), minimum and maximum values excluding outliers (whiskers)). **e**, Normalized wound-induced callus area in wild type, *pin2, pin3,4,7*, and *abcb1,19* under low water conditions (n = 20, 30, 19, 20, 20, 20 (left to right) independent explants; two-tailed Student’s t-test; box plot details as in (d)). **f**, Regeneration frequencies in *pin1* and *35S::PIN1-GFP* at high (0.75% agar) and low (2% agar/non-touch medium) water conditions (n = 20, 20, 21, 20, 19, 19 (left to right) independent explants; mean). **g**, Regeneration frequencies in wild type and *35S::PIN1-GFP* under intermediate water conditions (1.5% agar) (n = 14 (wild type), 16 (*35S::PIN1-GFP*) independent explants; mean). **h**, Regeneration frequencies in *pin2*, and *pin3,4,7* mutants at high and low water conditions (n = 20, 22, 20, 19, 20 20 independent explants; mean). DNRR= *de novo* root regeneration, WIC=wound-induced callus. Scale bar: 100 µm. The look-up table in **c** displays the signal intensity range used for **b** and **c**. Confocal experiments (**b**, **c**) were independently performed 3 times with similar results.

Our previous observations found that high water promoted a shift from proximal to distal auxin response maxima, so we investigated the basis for this. Using the DR5-VENUS reporter, we observed that ethylene precursor (ACC) and jasmonic acid (MeJA) treatments resulted in an auxin maxima that was farther from the wound site and more concentrated, similar to that observed during high-water conditions (Figs. 5a and 6a,b). MeJA treatments also reduced the SHY2-VENUS signal, similar to what occurred during high-water conditions (Extended Data Figs. 8c and 9a,b). We next investigated how stress hormones impact spatial auxin distribution, focusing on auxin transport. We found that genes encoding auxin efflux and influx carriers were differentially expressed under different water conditions (Extended Data Fig. 10a–c). In particular, *PINFORMED1–CFP* (*PIN1–CFP*) accumulated at the root primordium initiation site early in regeneration under conditions of high water availability (Fig. 6c). Testing several auxin transport mutants revealed that *pin1* and the *AUXIN RESISTANT 1/LIKE-AUX1* (*AUX1/LAX*) mutants *aux1,lax1,2,3* exhibited substantial defects in DNRR, with minimal or no impact on WIC formation (Fig. 6d,e and Extended Data Fig. 10d–h). Overexpression of *PIN1* (*35S::PIN1–GFP*), which interferes with polar auxin transport^34^, also caused strong defects in DNRR but had no significant effect on WIC formation, highlighting an important role for PIN1-dependent polar auxin transport in fate determination (Fig. 6d,e and Extended Data Fig. 10f,g). Furthermore, treatment with the auxin transport inhibitor *N*-1-naphthylphthalamic acid (NPA) showed that DNRR was more sensitive to auxin transport perturbation than WIC, although both regeneration processes appeared to require a basal level of auxin transport (Fig. 6f). We also tested MeJA treatments and found that they rapidly induced PIN1 expression at the root primordium initiation site (Fig. 6g,h). Our findings suggest that stress hormones, including jasmonic acid, regulate spatial auxin distribution via PIN1, helping to narrow and define distal auxin maxima that promote DNRR (Fig. 6i).

## Discussion

During wound healing, various regeneration processes can become activated. Here, we uncovered a critical role for the vascular cambium in WIC formation. Previous work has observed divisions in the vascular stele and pericycle upon early WIC formation^23^, and our findings broaden and expand this work to describe key molecular players and cell types involved in this process. Importantly, WIC formation appeared to inhibit DNRR formation, suggesting a trade-off between developmental fates consistent with what has been proposed for xylem pole pericycle cells giving rise to either lateral roots or cambium^35^. When tissues detach from a growing plant, they can lose water, nutrients, growth signals and positional cues, necessitating their reliance on internal and external stimuli for survival and adaptation. Our study reveals external water availability as a key factor determining regeneration fates in wounded tissues, seemingly playing a greater role than nutrients and physical contact observed in other developmental processes^36–38^. Mechanical pressure along with developmental age and positional information all impact cell fate changes during restorative regeneration^39,40^. However, our study revealed that physical force or touch alone did not determine regeneration fates in detached tissues. Differences in regeneration observed between intact and detached tissues could be due to detached or poorly connected tissues facing a restricted environment from lost connections, missing positional information and increased dehydration unlike intact tissues undergoing restorative regeneration that are affected by physical cues^39,40^. Our conclusions also contrast those from a previous study that did not test the role of water availability at the wound site itself^15^; such differences can be reconciled given our finding regarding the importance of water at the wound site. Consequently, we propose that detached tissues rely on water signals, important for sustaining cell viability, to determine their regeneration fates leading to the regeneration of either root for water uptake or pluripotent callus for wound sealing. Severe damage across multiple cell layers in mature tissues could also respond to water availability in fate determination, as observed in air layering. Thus, water signals appear to exert greater influence over other signals on detached and damaged tissues.

Water availability is also crucial for root branching in lateral root hydropatterning^3^. Water dictates a single developmental fate in lateral root hydropatterning, with irreversible changes occurring in as little as 20 min of deficiency^1^. By contrast, here we show that water determines a balance between two regeneration fates operating through distinct pathways, offering some reversibility. This underscores the greater flexibility of our findings with regeneration. Furthermore, regenerating tissues respond to fluctuating water availability rather than mere presence or absence, contrasting with xerobranching, an extreme form of hydropatterning, wherein water absence represses root branching^3,41^. We found that water levels influenced the distinct spatial distribution of auxin response maxima in regeneration pathways by coordination between auxin transporters and the stress hormones ethylene and jasmonic acid. We uncovered that transient elevations in these stress hormones were crucial for regeneration, consistent with previous work^28,30^. However, overproduction negatively affected regeneration fates (Fig. 4e,f), similar to the detrimental effects on regeneration previously reported with higher doses or constant treatments of stress hormones^30,42^, probably triggering defence pathways^43^. This suggests a potential trade-off between regeneration and defence^44^, where the initial surge in stress hormones promotes regeneration, yet prolonged activation may trigger defensive responses. Such defence responses could be an extreme manifestation of adaptive responses. However, further investigation is required to understand this correlation and explore whether extreme water levels or water deprivation also trigger a defence-like response, potentially linking to mechanisms such as xerobranching. Altogether, our data provide mechanistic insight into how spatial auxin distribution drives fate determination with water availability and stress hormones acting as key regulators early in the process (Fig. 6i), opening new research avenues.

The role of water availability in conventional tissue culture protocols has been largely overlooked, with attention focused instead on optimizing nutrients and hormones. Our findings highlight the crucial role of water in regeneration, both in hormone-free and in hormone-containing tissue culture media. Our study provides mechanistic insights into how water availability influences regeneration, offering a simple yet potentially effective tool for enhancing regeneration in tissue culture and plant transformation. Adjusting water availability in media could significantly improve regeneration (Fig. 3i and Extended Data Fig. 4f), particularly in species for which existing protocols are suboptimal. Moreover, fine-tuning stress hormones such as abscisic acid, ethylene and jasmonic acid through transient exposure or low concentrations offer additional potential for improving regeneration. This approach is particularly relevant in somatic embryogenesis, where use of stress hormones is common^45,46^, as well as in hormone-induced pluripotent callus formation, where short-term tissue submergence via an ethylene-mediated pathway helps to promote callus formation^47^. Taken together, our research sheds light on the origins of WIC and paves the way for major advancements in agricultural regeneration techniques by optimizing water availability and hormonal balance to improve outcomes across various plant species and tissue types.

## Methods

### Plant materials and growth conditions

*Arabidopsis thaliana* ecotype Columbia-0 (Col-0) was used throughout as the wild type, unless otherwise indicated. Tomato (*Solanum lycopersicum*) variety Moneymaker was used. All *Arabidopsis* mutants and transgenic lines used in this study have been previously described, and the details are listed in Supplementary Table 1. *Arabidopsis* seeds were surface-sterilized with 70% ethanol for 10 min and dried on sterilized filter paper. The seeds were then plated on half-strength Murashige and Skoog (MS) medium (pH 5.7–5.75) containing half-strength MS salts (including vitamins), 0.5% sucrose, 0.25 g l^−1^ 2-(*N*-morpholino)ethanesulfonic acid (MES) and 0.8% (w/v) plant agar. After 2 days of stratification at 4 °C in the dark, seedlings were grown vertically at 22 °C under a 16-h light (110 μE intensity) and 8-h dark photoperiod.

Tomato seeds were surface sterilized with 75% bleach for 10–15 min, washed five times in water and plated on MS medium (pH 5.7–5.75) containing half-strength MS salts (including vitamins), 0.5% sucrose, 0.25 g l^−1^ MES and 0.8% (w/v) plant agar. The plates were then transferred to a growth cabinet at 25 °C with a 16-h light and 8-h dark photoperiod.

### Wounding and regeneration assays

Cotyledon, leaf and hypocotyl explants were used for wound-induced regeneration in both *Arabidopsis* and tomato. Cotyledons with approximately 0.5-mm petioles were carefully detached from 7-day-old seedlings using microscissors. Hypocotyls were wounded above the root–hypocotyl junction in 7-day-old seedlings. For leaf wounding, the first pair of leaves with petioles (0.5 mm) were detached from 10-day-old seedlings. The detached explants were subsequently transferred to hormone-free Gamborg’s B5 medium (pH 5.7) containing Gamborg’s B5 salts (including vitamins), 2% glucose, 0.5 g l^−1^ MES and 0.75% (w/v) plant agar unless otherwise indicated. The explants were oriented with their wound site either touching the medium (abaxial side facing the medium for leaf and cotyledon) or not touching the medium (adaxial side facing the medium for leaf and cotyledon), as described previously^15^. The explants were cultured horizontally for regeneration under the same growth conditions as mentioned above. Regeneration responses were analysed between 6 days and 10 days after wounding. For the regeneration response on parafilm, the detached leaf explants were oriented with their wound site in contact with a parafilm strip placed on B5 medium. For the sandwich assay, the wound site of the petiole was positioned between a layer of parafilm and a thin layer of 0.75% agar, or between two layers of parafilm with an agar layer on top, forming a sandwich-like set-up. For the regeneration response to water availability, the detached leaf explants were transferred onto the B5 medium with varying concentrations of agar (0.375%, 0.75%, 1.2%, 1.5%, 2% and 3% plant agar (Duchefa Biochemie)). The water potential of the agar plates was calculated using the following formula after measuring the water activity (aw) and temperature (*T*) of the different agar media with a water activity meter (AquaLab Pre)

$${\rm{Water}}\; {\rm{potential}}=\frac{\left(8.314\right.\times \left(274+T\right)\times {\mathrm{ln}}({\mathrm{aw}})}{18}.$$

Water potential measurements were made in triplicate for each agar concentration and averaged.

For regeneration on nutrient-free medium, the detached leaves were cultured on a medium containing only agar without added nutrients. For hormone-induced callus (HIC) formation without wounding, 10-day-old whole seedlings were transferred onto a B5 medium supplemented with 2 μg ml^−1^ of 2,4-dichlorophenoxyacetic acid and 0.05 μg ml^−1^ of kinetin. After 5 days of culture, the petiole calli were collected for confocal imaging. For hormone-induced root regeneration in tomato, fully developed cotyledon was cut to a size of 0.5 cm and cultured on RIM (pH 5.7) containing Gamborg’s B5 salts (including vitamins), 2% glucose, 0.5 g l^−1^ MES, 0.5 mg l^−1^ indole-3-acetic acid and varying agar concentrations (0.35–1.5% plant agar). Cultures were maintained under long day conditions at 25 °C, and the number of roots per explant was counted after 3 weeks. For HIC formation in tomato, cotyledon cut pieces of 0.5 cm were cultured on CIM (pH 5.8) containing MS salts (including vitamins), 30 g l^−1^ sucrose, 1.5 mg l^−1^ zeatin and varying agar concentrations (0.35–1.5% plant agar), following a modified protocol^48^. Cultures were kept under long day conditions at 25 °C and subcultured every 2 weeks. The tissue area was measured after 26 days. For air layering in tomato, the stem was wounded using a sharp blade in the greenhouse. The wounded area was then wrapped with plastic film containing 5 g of either dry or wet soil, and tissue regeneration was monitored after 2 weeks.

### Chemical treatments

For the inducible overexpression of *LBD11* and *WOX4*, *35S::XVE≫LBD11* and *35S::XVE≫WOX4* seedlings were pretreated with B5 medium containing 5 µM oestradiol for 6 h before wounding. After wounding, tissues were placed on the same medium, either touching or not touching it. An equivalent volume of dimethyl sulfoxide (DMSO) was added to the B5 medium for the mock treatment. For *PLT2* overexpression, *35S::PLT2-GR* petiole tissues were cultured on B5 medium with 5 µM dexamethasone with the wound site not touching the medium. Ethanol was used for the mock treatment. For auxin treatments, petiole tissues were cultured on B5 medium with 0.2 µM NAA or DMSO (mock) under conditions of high (0.75% agar) or intermediate (1.5% agar) water availability. For inhibition of polar auxin transport, 1 µM or 2 µM NPA or DMSO (mock) was added to the B5 medium and the petiole tissues were cultured under high- or intermediate-water conditions. For ethylene precursor and jasmonic acid treatments, 4 µM ACC or 5 µM MeJA was added to the B5 medium, with water or ethanol, respectively, as the mock. Petiole explants were cultured for 4, 24 or 43 h under high- or intermediate-water conditions and then transferred onto hormone-free B5 medium under the same water conditions. For inhibition of ethylene activity, 10 µM silver nitrate (AgNO3) or water (mock) was added to the B5 medium and the petiole tissues were cultured under high- or intermediate-water conditions. For the NAA + AgNO_3_ treatment, 0.2 µM NAA and 10 µM AgNO_3_ were added together to the medium, and the petiole tissues were cultured under intermediate-water conditions.

### Tissue fixation and clearing for confocal imaging

The regenerating tissues with various fluorophores were fixed, cleared and then stained for cell walls before confocal imaging using a protocol that we modified from the previously published method^49^. In brief, tissues were fixed with 4% paraformaldehyde in 1× phosphate-buffered saline (PBS) buffer with 0.1% Triton X-100 for 1 h at room temperature, including a 10-min vacuum application. After fixation, the tissues were washed twice in 1× PBS, followed by treatment with absolute methanol and then absolute ethanol for 30 min each. Subsequently, after a wash with 1× PBS, the tissues underwent clearing in ClearSee solution (10% xylitol, 15% sodium deoxycholate and 25% urea) at room temperature for 2–3 days. The tissues were then stained in 0.1% Calcofluor-white in ClearSee solution for 30–60 min, followed by destaining in ClearSee for 15 min.

### Microscopy

Confocal images were captured using a Zeiss LSM780 with a 20× dry objective (numerical aperture 0.8). The pixel format was set to either 512 × 512 or 1,024 × 1,024 (for high-resolution images). A bidirectional scan was configured with a scan speed of 8, using line averaging for 4. The following wavelength settings were used for imaging various fluorophores: for Calcofluor-white imaging, excitation wavelength (ex.) 405 nm and emission (em.) 414–471 nm; for CFP, ex. 458 nm and em. 464–499 nm; for GFP, ex. 488 nm and em. 491–519 nm; for YFP and Venus, ex. 514 nm and em. 518–540 nm. The laser power and pinhole were adjusted on the basis of fluorescence brightness to prevent signal saturation or bleaching. The gain was set to 1. Imaging of CFP, GFP and YFP/Venus was conducted using a ChS1 (high-sensitivity GaAsP PMTs) detector. The settings remained unchanged throughout each experiment where fluorescent signal comparison was needed. Figure 6g imaging utilized a Leica Stellaris 5 confocal microscope with a 20× glycerol objective (numerical aperture 0.75) and HyD detectors. Images were acquired at a resolution of 512 × 512 pixels using bidirectional scanning at 400 Hz with a line average of 4. Calcofluor-white was excited at 405 nm with emission collected between 420 nm and 460 nm at 1.68% laser intensity. CFP fluorescence was excited at 448 nm with emission collected between 460 nm and 490 nm at 1% laser intensity. Sequential scanning was used. Bright-field images of regenerating callus and de novo roots were captured with a Leica M205FA stereomicroscope.

### Image analysis and data processing

The images were analysed using FIJI software (https://fiji.sc). The callus area was measured by the freehand tool in FIJI. Images were sometimes rotated for better orientation. The brightness of the fluorescence signal was adjusted for improved visualization, and these adjustments were consistently equal across various timepoints or treatments. Images were then annotated and arranged in Affinity Designer (version 1.9.1). All cartoons in the figures were created using Inkscape software version 1.3.2 (091e20e, 2023-11-25) (https://inkscape.org/).

### Reverse transcription quantitative PCR assays

*Arabidopsis* leaf petioles from wild-type and *lbd1,11* mutant plants were wounded and cultured for regeneration with the wound site not in contact with the medium. Approximately 0.5 mm of tissue from the wound site was collected 4 days after wounding. Total RNA was extracted using the Roti-Prep RNA MINI Kit, and the RNA samples were quantified with a NanoDrop ND-1000 spectrometer (Thermo Fisher Scientific). For cDNA synthesis, 500 ng of total RNA was reverse-transcribed using the Maxima First Strand cDNA Synthesis Kit, which includes oligo(dT) and random hexamer primers. Quantitative PCR (qPCR) was carried out with the iCycler iQ Real-Time PCR detection system in 10-μl reaction volumes. Each reaction contained 5 μl of 2× Maxima SYBR Green qPCR/ROX Master Mix, 0.75 μM of both forward and reverse primers, and 2 μl of diluted cDNA. The quantitative PCR with reverse transcription (RT–qPCR) protocol included an initial denaturation at 95 °C for 10 min, followed by 40 cycles of 95 °C for 10 s and 60 °C for 30 s. A melt curve analysis was performed to ensure specificity by confirming the absence of off-target amplification. Gene expression levels were quantified using the 2^−ΔΔCT^ method, with normalization to the ACTIN2 reference gene. The list of primers is provided in Supplementary Table 2.

### Transcriptomic analyses

Approximately 0.5 mm of tissue from the wound site of *Arabidopsis* wild-type leaf petioles treated with conditions of high (0.75% agar) or low (2% agar) water availability was collected at 0, 3, 6, 24 and 72 h after wounding. Samples were collected in three biological replicates, with approximately 60 petioles combined per sample. Total RNA was extracted using the Roti-Prep RNA MINI Kit according to the manufacturer’s instructions. mRNA library preparation (poly-A enrichment) and sequencing (NovaSeq X Plus in 150-bp paired-end) were performed at Novogene.

For RNA-seq analyses, low-quality reads were filtered out using Fastp^50^, and the cleaned reads were mapped to the *Arabidopsis* reference genome (TAIR10) with Hisat2^51^. The read counts were determined using HTSeq^52^. Differentially expressed genes (DEGs) were identified with the DESeq2 R package^53^, using a threshold *q* value <0.05. Genes with a *q* value <0.05 and an absolute log_2_(fold change) >1 were considered to exhibit statistically significant expression differences between samples. At each timepoint, the low-water condition served as the reference. For each sample comparison, the time 0 sample was used as the reference. Gene expression patterns under different water conditions were analysed using the Mfuzz package^54^ in R. Initially, DEGs from all timepoints were compiled into a single list for analysis. Time 0 samples were also included to identify gene expression patterns under high- and low-water conditions based on the list of DEGs. Gene Ontology (GO) annotation was conducted using the clusterProfiler package^55^. Enriched GO terms were identified with an adjusted *P* value of less than 0.05, an enrichment fold change greater than 2 and a DEG count above 10. The lists of DEGs and GO annotations are provided in Supplementary Data 1 and 2. Heatmaps of hormone-responsive genes^23,56^ were generated using R (v4.3.3) with the pheatmap package.

### Statistical analysis

Statistical analysis was conducted using Excel version 16.77.1 or RStudio version 4.41.1. A two-tailed Student’s *t*-test was performed between two groups within Excel, and Kruskal–Wallis analysis was performed in RStudio. Statistical significance was determined by *P* values <0.05.

### Reporting summary

Further information on research design is available in the Nature Portfolio Reporting Summary linked to this article.

## Supplementary information

Supplementary Information Supplementary Tables 1 and 2.

Reporting Summary

Supplementary Data 1 DEGs identified under high- and low-water conditions during regeneration.

Supplementary Data 2 GO enrichment analysis of transcriptome clusters under high- and low-water conditions during regeneration.

## Data Availability

All data are accessible in the Article and its Supplementary Information and extended data. Transcriptomic data reported in this Article are deposited in the NCBI Sequence Read Archive (SRA) with BioProject ID PRJNA1129885 and are available at https://www.ncbi.nlm.nih.gov/bioproject/PRJNA1129885. This study did not generate original code.
