# Supplementary material for: Water availability positions auxin response maxima to determine plant regeneration fates
Source: Nat Plants. 2025 Jul 4;11(7):1367–79. doi: 10.1038/s41477-025-02029-2 (PMC12283381; doi:10.1038/s41477-025-02029-2)
Supplement: Supplementary file 1 — Supplementary Tables 1 and 2. [file 41477_2025_2029_MOESM1_ESM.pdf]

nature plants

Supplementary information

https://doi.org/10.1038/s41477-025-02029-2

Water availability positions auxin response maxima to determine plant regeneration fates

In the format provided by the authors and unedited

Supplementary Table1: List of Arabidopsis mutants and transgenic lines used in this study.

Genotype	Background	Reference
arf6-2	Columbia	Nagpal et al.[1]
arf7-1	Columbia	Okushima et al.[2]
arf8-3	Columbia	Nagpal et al.[1]
arf7-1,arf19-1	Columbia	Okushima et al [2]
aux1,lax1,lax2,lax3	Columbia	Bainbridge et al.[3]
ein2-1	Columbia	Guzmán and Ecker [4]
eto2	Wassilewskija	Vogel et al. [5]
eto3	Columbia	Woeste et al. [6]
etr1-1	Columbia	Bleecker et al. [7]
lbd1,lbd11	Columbia	Ye et al. [8]
lbd1,lbd3,lbd4,lbd11	Columbia	Ye et al. [8]
lbd16-1	Columbia	Okushima et al.[9]
pin1-613	Columbia	Bennett et al.[10]
pin2 (eir1-1)	Columbia	Roman et al.[11]
pin3-3,pin4-3,pin7-1	Columbia	Blilou et al.[12]
abcb1-100,abcb19-101	Columbia	Bennett et al.[10]
slr-1	Columbia	Fukaki et al.[13]
shy2-31	Landsberg *erecta*	Knox et al.[14]
wox4-1	Columbia	Hirakawa et al. [15]
wox14-1	Columbia	Etchells et al. [16]
wox4-1,wox14-1	Columbia	Etchells et al. [16]
wox13-2,wox14-1	Columbia	Sakakibara et al. [17]
yuc2,yuc5,yuc8,yuc9	Columbia	(Müller-Moulé et al. [18]
yuc1D	Columbia	Weigel et al. [19]
35S::XVE>>LBD11	Columbia	Ye et al.[8]
35S::LBD16	Columbia	Orosa-Puente et al. [20]
35S::PIN1-GFP	Columbia	Marhavý et al.[21]
35S::PLT2-GR	Columbia	Galinha et al.[22]
35S::WOX11	Columbia	Liu et al.[23]
35S::XVE>>WOX4	Columbia	Zhang et al.[24]
$ACS6_{pro}$::NLS-3xVenus	Columbia	Marhavý et al.[25]
DR5rev:3xVenus-N7	Columbia	Heisler et al.[26]
$JAZ10_{pro}$::NLS-3xVenus	Columbia	Marhavý et al.[25]
$LBD1_{pro}$::erYFP	Columbia	Ye et al.[8]
$LBD11_{pro}$::erYFP	Columbia	Ye et al.[8]

LBD16pro::LBD16-GFP	Columbia	Orosa-Puente et al.[20]
PIN1pro::PIN1-CFP	Landsberg *erecta*	Gordon et al.[27]
PIN2pro::PIN2-GFP	Columbia	Abas et al.[28]
PIN3pro::PIN3-GFP	Columbia	Žádníková et al.[29]
PLT2pro::PLT2-YFP	Columbia	Mähönen et al.[30]
PXYpro:erECFP	Columbia	Agusti et al. [31]
SHY2/IAA2pro::NLS-3xmVENUS	Columbia	Vermeer et al.[32]
WOX4pro:erEYFP	Columbia	Suer et al.[33]
WOX11pro:H2B-eGFP	Columbia	Zhai and Xu[34]
YUC4pro::NLS-3xGFP	Columbia	Robert et al.[35]

Supplementary Table2. List of primers used in this study

Primers Used for Quantitative Real-Time PCR

Gene	Forward primer	Reverse primer
ACT2	5’-ACATTGTGCTCAGTGGTGGA	5’-CTGAGGGAAGCAAGAATGGA
LBD16	5'-TCCATGATCGATGTGAAGCTGTCG	5'-TGTGATTGCAAGAAAGCCACCTG
WOX11	5’-TTCAATACCAACAAGGGGCT	5’-CCTGAGGAATGCACCAAAACC

Supplementary references

1 Nagpal, P. *et al.* Auxin response factors ARF6 and ARF8 promote jasmonic acid production and flower maturation. *Development* **132**, 4107-4118 (2005). https://doi.org:10.1242/dev.01955

2 Okushima, Y. *et al.* Functional Genomic Analysis of the AUXIN RESPONSE FACTOR Gene Family Members in Arabidopsis thaliana: Unique and Overlapping Functions of ARF7 and ARF19 *The Plant Cell* **17**, 444-463 (2005). https://doi.org:10.1105/tpc.104.028316

3 Bainbridge, K. *et al.* Auxin influx carriers stabilize phyllotactic patterning. *Genes Dev* **22**, 810-823 (2008). https://doi.org:10.1101/gad.462608

4 Guzmán, P. & Ecker, J. R. Exploiting the triple response of Arabidopsis to identify ethylene-related mutants. *Plant Cell* **2**, 513-523 (1990). https://doi.org:10.1105/tpc.2.6.513

5 Vogel, J. P., Woeste, K. E., Theologis, A. & Kieber, J. J. Recessive and dominant mutations in the ethylene biosynthetic gene ACS5 of Arabidopsis confer cytokinin insensitivity and ethylene overproduction, respectively. *Proc Natl Acad Sci U S A* **95**, 4766-4771 (1998). https://doi.org:10.1073/pnas.95.8.4766

6 Woeste, K. E., Ye, C. & Kieber, J. J. Two Arabidopsis mutants that overproduce ethylene are affected in the posttranscriptional regulation of 1-aminocyclopropane-

1-carboxylic acid synthase. *Plant Physiol* **119**, 521-530 (1999). https://doi.org:10.1104/pp.119.2.521
7 Bleecker, A. B., Estelle, M. A., Somerville, C. & Kende, H. Insensitivity to Ethylene Conferred by a Dominant Mutation in <i>Arabidopsis thaliana</i>. *Science* **241**, 1086-1089 (1988). https://doi.org:doi:10.1126/science.241.4869.1086
8 Ye, L. *et al.* Cytokinins initiate secondary growth in the Arabidopsis root through a set of LBD genes. *Current Biology* **31**, 3365-3373.e3367 (2021). https://doi.org:https://doi.org/10.1016/j.cub.2021.05.036
9 Okushima, Y., Fukaki, H., Onoda, M., Theologis, A. & Tasaka, M. ARF7 and ARF19 Regulate Lateral Root Formation via Direct Activation of LBD/ASL Genes in Arabidopsis. *The Plant Cell* **19**, 118-130 (2007). https://doi.org:10.1105/tpc.106.047761
10 Bennett, T. *et al.* The Arabidopsis MAX Pathway Controls Shoot Branching by Regulating Auxin Transport. *Current Biology* **16**, 553-563 (2006). https://doi.org:https://doi.org/10.1016/j.cub.2006.01.058
11 Roman, G., Lubarsky, B., Kieber, J. J., Rothenberg, M. & Ecker, J. R. Genetic analysis of ethylene signal transduction in Arabidopsis thaliana: five novel mutant loci integrated into a stress response pathway. *Genetics* **139**, 1393-1409 (1995). https://doi.org:10.1093/genetics/139.3.1393
12 Blilou, I. *et al.* The PIN auxin efflux facilitator network controls growth and patterning in Arabidopsis roots. *Nature* **433**, 39-44 (2005). https://doi.org:10.1038/nature03184
13 Fukaki, H., Tameda, S., Masuda, H. & Tasaka, M. Lateral root formation is blocked by a gain-of-function mutation in the SOLITARY-ROOT/IAA14 gene of Arabidopsis. *The Plant Journal* **29**, 153-168 (2002). https://doi.org:https://doi.org/10.1046/j.0960-7412.2001.01201.x
14 Knox, K., Grierson, C. S. & Leyser, O. AXR3 and SHY2 interact to regulate root hair development. *Development* **130**, 5769-5777 (2003). https://doi.org:10.1242/dev.00659
15 Hirakawa, Y., Kondo, Y. & Fukuda, H. TDIF Peptide Signaling Regulates Vascular Stem Cell Proliferation via the WOX4 Homeobox Gene in Arabidopsis *The Plant Cell* **22**, 2618-2629 (2010). https://doi.org:10.1105/tpc.110.076083
16 Etchells, J. P., Provost, C. M., Mishra, L. & Turner, S. R. WOX4 and WOX14 act downstream of the PXY receptor kinase to regulate plant vascular proliferation independently of any role in vascular organisation. *Development* **140**, 2224-2234 (2013). https://doi.org:10.1242/dev.091314
17 Sakakibara, K. *et al.* WOX13-like genes are required for reprogramming of leaf and protoplast cells into stem cells in the moss Physcomitrella patens. *Development* **141**, 1660-1670 (2014). https://doi.org:10.1242/dev.097444
18 Müller-Moulé, P. *et al.* YUCCA auxin biosynthetic genes are required for Arabidopsis shade avoidance. *PeerJ* **4**, e2574 (2016). https://doi.org:10.7717/peerj.2574
19 Weigel, D. *et al.* Activation Tagging in Arabidopsis1. *Plant Physiology* **122**, 1003-1014 (2000). https://doi.org:10.1104/pp.122.4.1003
20 Orosa-Puente, B. *et al.* Root branching toward water involves posttranslational modification of transcription factor ARF7. *Science* **362**, 1407-1410 (2018). https://doi.org:doi:10.1126/science.aau3956

21 Marhavý, P. *et al.* Cytokinin Controls Polarity of PIN1-Dependent Auxin Transport during Lateral Root Organogenesis. *Current Biology* **24**, 1031-1037 (2014). https://doi.org:https://doi.org/10.1016/j.cub.2014.04.002
22 Galinha, C. *et al.* PLETHORA proteins as dose-dependent master regulators of Arabidopsis root development. *Nature* **449**, 1053-1057 (2007). https://doi.org:10.1038/nature06206
23 Liu, J. *et al.* WOX11 and 12 are involved in the first-step cell fate transition during de novo root organogenesis in Arabidopsis. *Plant Cell* **26**, 1081-1093 (2014). https://doi.org:10.1105/tpc.114.122887
24 Zhang, J. *et al.* Transcriptional regulatory framework for vascular cambium development in Arabidopsis roots. *Nature Plants* **5**, 1033-1042 (2019). https://doi.org:10.1038/s41477-019-0522-9
25 Marhavý, P. *et al.* Single-cell damage elicits regional, nematode-restricting ethylene responses in roots. *The EMBO Journal* **38**, e100972 (2019). https://doi.org:https://doi.org/10.15252/embj.2018100972
26 Heisler, M. G. *et al.* Patterns of Auxin Transport and Gene Expression during Primordium Development Revealed by Live Imaging of the Arabidopsis Inflorescence Meristem. *Current Biology* **15**, 1899-1911 (2005). https://doi.org:https://doi.org/10.1016/j.cub.2005.09.052
27 Gordon, S. P. *et al.* Pattern formation during de novo assembly of the Arabidopsisshoot meristem. *Development* **134**, 3539-3548 (2007). https://doi.org:10.1242/dev.010298
28 Abas, L. *et al.* Intracellular trafficking and proteolysis of the Arabidopsis auxin-efflux facilitator PIN2 are involved in root gravitropism. *Nature Cell Biology* **8**, 249-256 (2006). https://doi.org:10.1038/ncb1369
29 Žádníková, P. *et al.* Role of PIN-mediated auxin efflux in apical hook development of Arabidopsis thaliana. *Development* **137**, 607-617 (2010). https://doi.org:10.1242/dev.041277
30 Mähönen, A. P. *et al.* PLETHORA gradient formation mechanism separates auxin responses. *Nature* **515**, 125-129 (2014). https://doi.org:10.1038/nature13663
31 Agusti, J. *et al.* Strigolactone signaling is required for auxin-dependent stimulation of secondary growth in plants. *Proceedings of the National Academy of Sciences* **108**, 20242-20247 (2011). https://doi.org:doi:10.1073/pnas.1111902108
32 Vermeer, J. E. *et al.* A spatial accommodation by neighboring cells is required for organ initiation in Arabidopsis. *Science* **343**, 178-183 (2014). https://doi.org:10.1126/science.1245871
33 Suer, S., Agusti, J., Sanchez, P., Schwarz, M. & Greb, T. WOX4 imparts auxin responsiveness to cambium cells in Arabidopsis. *Plant Cell* **23**, 3247-3259 (2011). https://doi.org:10.1105/tpc.111.087874
34 Zhai, N. & Xu, L. Pluripotency acquisition in the middle cell layer of callus is required for organ regeneration. *Nature Plants* **7**, 1453-1460 (2021). https://doi.org:10.1038/s41477-021-01015-8
35 Robert, H. S. *et al.* Local auxin sources orient the apical-basal axis in Arabidopsis embryos. *Curr Biol* **23**, 2506-2512 (2013). https://doi.org:10.1016/j.cub.2013.09.039
