# Supplementary material for: Water availability positions auxin response maxima to determine plant regeneration fates
Source: Nat Plants. 2025 Jul 4;11(7):1367–79. doi: 10.1038/s41477-025-02029-2 (PMC12283381; doi:10.1038/s41477-025-02029-2)
Supplement: Supplementary file 2 — Reporting Summary [file 41477_2025_2029_MOESM2_ESM.pdf]

nature portfolio

Corresponding author(s): Charles Melnyk and Abdul Kareem

Last updated by author(s): May 6, 2025

Reporting Summary

Nature Portfolio wishes to improve the reproducibility of the work that we publish. This form provides structure for consistency and transparency in reporting. For further information on Nature Portfolio policies, see our Editorial Policies and the Editorial Policy Checklist.

Statistics

For all statistical analyses, confirm that the following items are present in the figure legend, table legend, main text, or Methods section.

n/a	Confirmed	
☐	☒	The exact sample size (*n*) for each experimental group/condition, given as a discrete number and unit of measurement
☐	☒	A statement on whether measurements were taken from distinct samples or whether the same sample was measured repeatedly
☐	☒	The statistical test(s) used AND whether they are one- or two-sided<br>*Only common tests should be described solely by name; describe more complex techniques in the Methods section.*
☒	☐	A description of all covariates tested
☒	☐	A description of any assumptions or corrections, such as tests of normality and adjustment for multiple comparisons
☐	☒	A full description of the statistical parameters including central tendency (e.g. means) or other basic estimates (e.g. regression coefficient) AND variation (e.g. standard deviation) or associated estimates of uncertainty (e.g. confidence intervals)
☐	☒	For null hypothesis testing, the test statistic (e.g. *F*, *t*, *r*) with confidence intervals, effect sizes, degrees of freedom and *P* value noted<br>*Give P values as exact values whenever suitable.*
☒	☐	For Bayesian analysis, information on the choice of priors and Markov chain Monte Carlo settings
☒	☐	For hierarchical and complex designs, identification of the appropriate level for tests and full reporting of outcomes
☐	☒	Estimates of effect sizes (e.g. Cohen's *d*, Pearson's *r*), indicating how they were calculated

Our web collection on statistics for biologists contains articles on many of the points above.

Software and code

Policy information about availability of computer code

Data collection	Fluorescent Imaging: Zeiss LSM780 and Leica Stellaris 5 confocal microscopes<br>Bright-field imaging: Leica M205FA stereo microscope<br>RT-qPCR: iCycler iQ Real-Time PCR<br>Arabidopsis gene sequences: TAIR10
Data analysis	1. R (version 4.0.2) was used for transcriptome analysis . Low-quality reads were filtered out using Fastp, and the cleaned reads were mapped to the Arabidopsis reference genome (TAIR10) with Hisat2. The read counts were determined using HTSeq. Differentially expressed genes (DEGs) were identified with the DESeq2 R package, using a threshold q-value < 0.05. Gene expression patterns under different water conditions were analyzed using the Mfuzz package in R. Gene ontology (GO) annotation was conducted using the clusterProfiler package. Enriched GO terms were identified with an adjusted p-value of less than 0.05, an enrichment fold change greater than 2, and a DEG count above 10. Heatmaps were generated using R (v4.3.3) with the pheatmap package<br>2.R studio (version 4.41.1) was used for Kruskal-Wallis analysis and for creating plots<br>3. Excel (version 16.77.1) was used for the Student's t-test analysis and for creating plots.<br>4. ImageJ (FIJI) (version2.3.0/1.53f) was used for image analysis<br>5. Affinity Designer (version 1.9.1) was used for image annotation and figure arrangement

For manuscripts utilizing custom algorithms or software that are central to the research but not yet described in published literature, software must be made available to editors and reviewers. We strongly encourage code deposition in a community repository (e.g. GitHub). See the Nature Portfolio guidelines for submitting code & software for further information.

Data

Policy information about availability of data

All manuscripts must include a data availability statement. This statement should provide the following information, where applicable:

- Accession codes, unique identifiers, or web links for publicly available datasets
- A description of any restrictions on data availability
- For clinical datasets or third party data, please ensure that the statement adheres to our policy

All data are accessible in the manuscript, supplementary materials, extended data, and source files. Transcriptomic data reported in this paper are deposited in the NCBI Sequence Read Archive (SRA) with BioProject ID PRJNA1129885. The transcriptomic data are available from SRA. This study did not generate original code.

Research involving human participants, their data, or biological material

Policy information about studies with human participants or human data. See also policy information about sex, gender (identity/presentation), and sexual orientation and race, ethnicity and racism.

Reporting on sex and gender	Not applicable
Reporting on race, ethnicity, or other socially relevant groupings	Not applicable
Population characteristics	Not applicable
Recruitment	Not applicable
Ethics oversight	Not applicable

Note that full information on the approval of the study protocol must also be provided in the manuscript.

Field-specific reporting

Please select the one below that is the best fit for your research. If you are not sure, read the appropriate sections before making your selection.

☒ Life sciences ☐ Behavioural & social sciences ☐ Ecological, evolutionary & environmental sciences

For a reference copy of the document with all sections, see nature.com/documents/nr-reporting-summary-flat.pdf

Life sciences study design

All studies must disclose on these points even when the disclosure is negative.

Sample size	Sample size was determined according to similar studies in the field (Zhang et al., 2019, DOI: 10.1038/s41477-019-0408-x)
Data exclusions	No data were excluded
Replication	All experiments have at least two biological replicates with similar results
Randomization	Seedlings or samples were always randomly distributed during growth and treatment. Most of the experimental findings were related to comparative analysis between wild type and mutant lines
Blinding	Blinding was not necessary for our experiments, because there was no individual/subjective bias in experimentation or data analysis.

Reporting for specific materials, systems and methods

We require information from authors about some types of materials, experimental systems and methods used in many studies. Here, indicate whether each material, system or method listed is relevant to your study. If you are not sure if a list item applies to your research, read the appropriate section before selecting a response.

Materials & experimental systems

n/a	Involved in the study	
☒	☐	Antibodies
☒	☐	Eukaryotic cell lines
☒	☐	Palaeontology and archaeology
☒	☐	Animals and other organisms
☒	☐	Clinical data
☒	☐	Dual use research of concern
☐	☒	Plants

Methods

n/a	Involved in the study	
☒	☐	ChIP-seq
☒	☐	Flow cytometry
☒	☐	MRI-based neuroimaging

Dual use research of concern

Policy information about dual use research of concern

Hazards

Could the accidental, deliberate or reckless misuse of agents or technologies generated in the work, or the application of information presented in the manuscript, pose a threat to:

No	Yes	
☒	☐	Public health
☒	☐	National security
☒	☐	Crops and/or livestock
☒	☐	Ecosystems
☒	☐	Any other significant area

Experiments of concern

Does the work involve any of these experiments of concern:

No	Yes	
☒	☐	Demonstrate how to render a vaccine ineffective
☒	☐	Confer resistance to therapeutically useful antibiotics or antiviral agents
☒	☐	Enhance the virulence of a pathogen or render a nonpathogen virulent
☒	☐	Increase transmissibility of a pathogen
☒	☐	Alter the host range of a pathogen
☒	☐	Enable evasion of diagnostic/detection modalities
☒	☐	Enable the weaponization of a biological agent or toxin
☒	☐	Any other potentially harmful combination of experiments and agents

Plants

Seed stocks	Mutants and transgenic lines used in this study were previously published and obtained from other researchers. The wox4-1 mutant was obtained from the Nottingham Arabidopsis Seed Centre (NASC) with NASC ID: N799841.
Novel plant genotypes	No new plant genotypes were generated for this study
Authentication	Known phenotypic characteristics were assessed for the mutants.
